# The transition state for coupled folding and binding of a disordered DNA binding domain resembles the unbound state

**DOI:** 10.1093/nar/gkae794

**Published:** 2024-09-24

**Authors:** Mikhail Kuravsky, Conor Kelly, Christina Redfield, Sarah L Shammas

**Affiliations:** Department of Biochemistry, University of Oxford, Oxford OX1 3QU, UK; Department of Biochemistry, University of Oxford, Oxford OX1 3QU, UK; Department of Biochemistry, University of Oxford, Oxford OX1 3QU, UK; Department of Biochemistry, University of Oxford, Oxford OX1 3QU, UK

## Abstract

The basic zippers (bZIPs) are one of two large eukaryotic families of transcription factors whose DNA binding domains are disordered in isolation but fold into stable α-helices upon target DNA binding. Here, we systematically disrupt pre-existing helical propensity within the DNA binding region of the homodimeric bZIP domain of cAMP-response element binding protein (CREB) using Ala-Gly scanning and examine the impact on target binding kinetics. We find that the secondary structure of the transition state strongly resembles that of the unbound state. The residue closest to the dimerization domain is largely folded within both unbound and transition states; dimerization apparently propagates additional helical propensity into the basic region. The results are consistent with electrostatically-enhanced DNA binding, followed by rapid folding from the folded zipper outwards. Fly-casting theory suggests that protein disorder can accelerate binding. Interestingly however, we did not observe higher association rate constants for mutants with lower levels of residual structure in the unbound state.

## Graphical Abstract

**Graphical Abstract:**
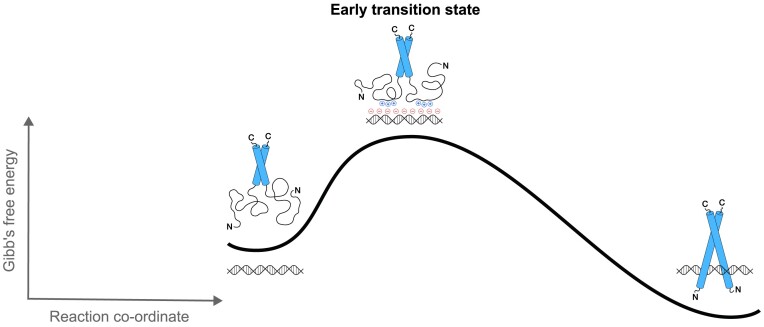

## Introduction

Eukaryotic transcription factors frequently contain intrinsically disordered regions that fold upon binding to target DNA sequences: the AT-hooks, bZIP and bHLH domains (1). In the absence of DNA, the basic DNA binding domains are dynamic but exhibit varying levels of pre-existing helical content (2–4). The existence of structures in the unbound state that resemble final folded structures accelerate binding processes if these are the ones selected for binding in a conformational selection mechanism (5). In contrast, if the domain only folds after binding in an induced fit mechanism then pre-existing structures may have little impact on association rates (6). Though not yet experimentally verified, protein disorder has even been speculated to be advantageous to association rates through a mechanism known as fly-casting (7). This mechanism involves region(s) of the extended disordered protein/region first binding to their partner with the rest of the protein being subsequently ‘reeled in’ and folding. Thus, the relevance of pre-existing structure in regions that fold upon binding could be intimately linked with folding mechanisms.

A protein's structure, or fold, is encoded in its amino acid sequence. But how are these folds achieved? Proteins have open to them an immense conformational space, yet natural proteins converge to their native states in sub-second timescales. A powerful experimental technique to probe protein folding mechanisms has undoubtedly been the Φ-value analysis (8). This mutation-based method was developed to describe the folding transition state. Conservative mutations are made to the amino acid sequence, and the effect on folding/unfolding rates is compared to the overall free energy change. The resulting Φ value, which typically lies in the range 0–1, describes the extent of eventual (native) contacts that the residue has acquired by the transition state (8). Over the last ten years this method has also been applied to investigate the coupled folding and binding of a handful of intrinsically disordered proteins/regions (IDPs/IDRs) with their partner proteins (9–21), however, the transition state for folding of a protein-DNA interaction has not yet been reported.

One protein containing a disordered DNA binding domain is the cAMP-response element binding protein (CREB). CREB is an ubiquitously expressed transcription factor regulating expression of a wide range of genes (22–24) through binding to cAMP-response element sites (CRE, consensus 5′-TGACGTCA-3′) in the proximal promoters of its targets (25,26). Phosphorylation at S133 within the Kinase Inducible Domain (KID) of CREB promotes interactions with its coactivator, CREB-binding protein (CBP) (27,28), enabling transcription by recruiting the core transcription machinery (29). CREB acts as a hub in a signalling network controlling proliferation (30), differentiation (31–33) and the survival of cells (34,35). Transcriptional activity of CREB plays a central role in a number of processes, including immune response (36), reproduction (37,38), long-term memory (39) and circadian rhythms (40).

**Figure 1.**
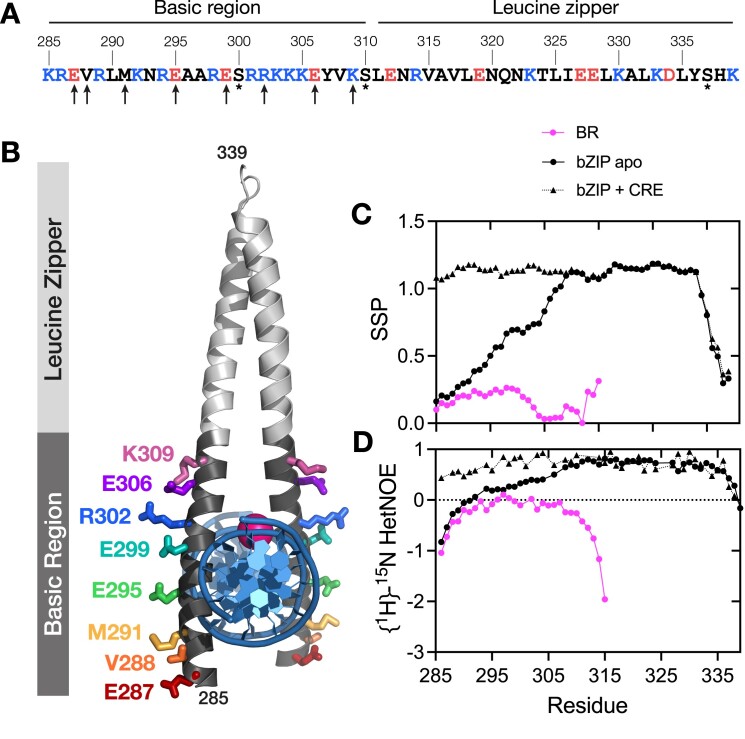
Structural description of CREB bZIP. (**A**) The sequence of the CREB _285_bZIP construct used in this study. Positively charged amino acids are shown in blue, negatively charged amino acids are shown in red. All cysteines were replaced with serines (asterisks). Eight solvent-exposed residues subjected to alanine and glycine substitutions are marked with arrows. (**B**) Structural positions of residues subjected to mutagenesis within CREB _285_bZIP-DNA complex. The Mg^2+^ ion is shown as magenta sphere. Derived from 1DH3. Calculated Secondary Structural Propensity (SSP) (**C**) and {^1^H}-^15^N heteronuclear NOE (**D**) of CREB _285_BR (pink circles), CREB _285_bZIP (black circles) and CREB _285_bZIP with CRE DNA (black triangles).

DNA binding by CREB is mediated by a C-terminal basic leucine zipper (bZIP) domain (41), part of a eukaryotic superfamily of transcription factors that has 56 human members (42). A typical bZIP binds to the DNA as a homo- or heterodimer. Each of the subunits consists of an N-terminal basic region (BR) and a C-terminal leucine zipper (LZ) (Figure 1A, B). The LZ promotes dimerization and is built of two amphipathic α-helices wrapped around each other in a parallel coiled-coil structure. The BR mediates DNA recognition and is enriched in positively charged amino acids (43). As shown in a number of X-ray structures, a bZIP assumes a chopstick-like structure of two uninterrupted α-helices grasping the DNA by the major groove (44–51). Although the bZIP domains have been studied for more than 30 years, there are few details on the mechanism of their binding to DNA. Here, we perform mutational scanning of the BR of homodimeric CREB bZIP to probe its binding mechanism and reveal the extent of helical formation within the transition state for binding.

## Materials and methods

### Reagents

NMR spectrometers equipped with 14.1 and 17.6 Tesla magnets (Oxford Instruments, Abingdon, UK) and Bruker Avance III HD consoles and 5mm TCI cryoprobes running Topspin 3.2 data acquisition software (Bruker BioSpin) (Bruker UK Ltd, Coventry, UK).

SX20 stopped-flow spectrophotometer (Applied Photophysics, Leatherhead, UK) equipped with 25 mm diameter 515 nm longpass filter (Thorlabs, Ely, UK, FGL515) or 25 mm diameter 305 nm longpass filter (Andover Corporation, Salem, USA, 305FG01-25), a 2.5 ml gastight 1002C series borosilicate glass stop syringe (Hamilton, Bonaduz, Switzerland, 81460), two 2.5 ml gastight 1002AD series drive borosilicate glass drive syringes (Hamilton, Bonaduz, Switzerland, 201250) and a 250 ul gastight borosolicate glass drive syringe (FlexFludics, Flex Fluidics LLC, Las Vegas, USA 100258TAP).

J-815 spectropolarimeter (Jasco, Heckmondwike, UK) and a 1 mm pathlength Hellma Suprasil quartz cuvette (Hellma UK Ltd, Southend-on-Sea, UK, 110-1-40).

Akta Purifier (Cytiva, Little Chalfont, UK) equipped with His-Trap HP Ni-NTA agarose column (Cytiva, Little Chalfont, UK, 17524802) or HiTrap SP HP cation exchange chromatography column (Cytiva, Little Chalfont, UK, 17115201).

Oligonucleotides, including AlexaFluor®488- labelled oligonucleotides (Life Technologies, Thermo Fisher Scientific, Horsham, UK)

Q5® Site-Directed Mutagenesis Kit (New England Biolabs Ltd, Hitchin, UK, E0552S)

QIAprep Spin Miniprep Kit (Qiagen Ltd, Manchester, UK, 27104)

Tween® 20 (Merck Life Science UK Ltd, Gillingham, UK, P9416)

TEV protease (New England Biolabs, Hitchin, UK, P8112S)

DTT (Merck Life Science UK Ltd, Gillingham, UK, D5545)

IPTG (Merck Life Science UK Ltd, Gillingham, UK, I6758)

Urea (Merck Life Science UK Ltd, Gillingham, UK, 51456)

Trizma® hydrochloride (Merck Life Science UK Ltd, Gillingham, UK, T3253)

Trizma® base (Merck Life Science UK Ltd, Gillingham, UK, T4661)

Sodium chloride (Merck Life Science UK Ltd, Gillingham, UK, S9888)

MES monohydrate (Merck Life Science UK Ltd, Gillingham, UK, 69889)

MES sodium salt (Merck Life Science UK Ltd, Gillingham, UK, M3885)

Magnesium chloride hexahydrate (Merck Life Science UK Ltd, Gillingham, UK, M2670)

M9 minimal media (di-sodium hydrogen orthophosphate anhydrous (CHE1878), potassium dihydrogen orthophosphate (CHE2948) and sodium chloride (CHE3320)

U-^13^C d-glucose (Cambridge Isotopes, Tewksbury, USA, CLM-1396)

^15^N NH_4_Cl (Cambridge Isotopes, Tewksbury, USA, NLM-467)

Tris-d_11_ solution (Sigma-Aldrich, Gillingham, UK, 486248)

Deuterium oxide, 99.9% (Sigma-Aldrich, Gillingham, UK, 151882)

Sodium azide (Merck Life Science UK Ltd, Gillingham, UK, 57HO514)

Pierce™ protease inhibitors (Thermo Scientific, Horsham, UK, A32963)

### Biological resources

pRSET-A vector (Invitrogen, Thermo Fisher Scientific, Waltham, USA, V35120)

*Escherichia coli* XL1-Blue Competent Cells (Agilent Technologies Inc, Santa Clara, USA, 200249)

*Escherichia coli* BL21(DE3)pLysS Competent Cells (Invitrogen, Thermo Fisher Scientific, Waltham, USA, C606003)

### Statistical analyses

Stated errors refer to standard deviation from replicate measurements, or from fits as outlined in the text. Calculated *P*-values are based on a two-tailed Pearson correlation analysis (*n* = 7).

### Novel programs, software, algorithms

None used.

### Web sites/data base referencing

http://agadir.crg.es/

https://www.ibbr.umd.edu/nmrpipe/

https://ccpn.ac.uk/software/analysisassign/

https://pound.med.utoronto.ca/∼JFKlab/Software/ssp_more.htm

https://www.graphpad.com/

https://quansoft.com/

https://www.holehouselab.com/tools

### Cloning

A synthetic gene for the bZIP domain of human CREB (_285_bZIP) corresponding to residues 285–339 of CREB-A (Uniprot P16220) was fused with the B1 domain of Streptococcal protein G (GB1) at the N-terminus and cloned into a pRSET-A vector. A tobacco etch virus (TEV) protease site was inserted between the GB1 and the bZIP sequence. The C300, C310 and C337 residues of _285_bZIP were mutated to serine as described in (52) to improve protein solubility without affecting its DNA binding properties. Plasmids encoding mutant versions of _285_bZIP were produced by point mutagenesis and verified by DNA sequencing. The monomeric basic region of CREB bZIP corresponding to residues 285–315 of CREB-A was generated by insertion of a stop codon. In the following, the numbering of amino acids is consistent with that of the longest CREB isoform (CREB-A).

### Protein expression and purification

Plasmids for the wild-type and mutant bZIP constructs were transformed into *Escherichia coli* BL21(DE3)pLysS. Cells were cultured at 37°C in 2xYT medium. After the OD_600_ reached 0.6–0.8, recombinant protein expression was induced by 1 mM IPTG, and culturing was continued for another 4 h. *E. coli* pellets were disrupted by sonication in 100 mM Tris–HCl pH 8.5, 50 mM NaCl (binding buffer). The lysate was cleared by centrifugation and loaded on a Ni-NTA agarose column (HisTrap HP; GE Healthcare). After rigorous washing with 100 mM Tris–HCl pH 8.5, 2 M NaCl to remove bacterial DNA, protein was eluted with a 0–2 M gradient of imidazole in the binding buffer. His6-GB1-tag was subsequently cleaved by TEV protease (3 mM, in the presence of 1 mM DTT) and separated from bZIP on a cation exchange column (HiTrap SP; GE Healthcare) using a 0–2 M gradient of sodium chloride. Finally, proteins were buffer exchanged into 10 mM MES pH 6.5, 150 mM NaCl, 10 mM MgCl_2_, 0.05% Tween-20 (biophysical buffer). Protein purity was confirmed by SDS-PAGE (Supplementary Figure S1) and electrospray ionization mass spectrometry (ESI-MS). The absence of nucleic acid contamination was assessed by measuring the 260/280 ratio to be 0.42. All protein constructs contain an additional glycine residue at the N-terminus as a remnant from TEV cleavage.

### Expression of isotopically labelled CREB

Isotope labelling used a modified version of the protocol by (53). In short, cells were grown in 1 l of 2× TY at 37°C, shaken at 180 rpm. Once OD_600_ ∼0.7 was reached, cells were pelleted at 5000 g for 15 min at room temperature, washed with 1× M9 minimal media and resuspended in 250 ml of 1× M9 minimal media containing FeCl_3_, MgSO_4_ and CaCl_2_ (1 mM respectively), supplemented with 4 g/l U-^13^C d-glucose (Cambridge Isotopes CLM-1396) and/or 1 g/l ^15^N NH_4_Cl (Cambridge Isotopes NLM-467). Cells were then incubated for 45 min at 37°C shaken at 180 rpm, induced with 1 mM IPTG and cultured for a further 4 h. Protein purification was then carried out as described above.

### Protein concentrations

The molar extinction coefficient of CREB _285_bZIP in biophysical buffer was determined as 2560 M^–1^ cm^–1^ by Gill and von Hippel method (54).

### Preparation of double-stranded DNA

The binding of CREB to a consensus-CRE-containing DNA sequence was examined using self-annealing CRE oligonucleotides (5′-CC**TGACGTCA**GCCCCC**TGACGTCA**GG-3′) prepared according to (55) and labelled with Alexa Fluor®488 at the 5′-terminus (Alexa Fluor®488-CREh). Dissociation kinetics were studied using a 14 bp double stranded competitor CRE DNA (5′-CC**TGACGTCA**TCCG-3′). Out-competition using identical (non-labelled) sequences results in additional phases in stopped-flow experiments that are also present without CREB. Finally, NMR studies utilized a symmetric CRE-containing sequence (5′-AGA**TGACGTCA**TCT-3′). Annealing was performed by mixing the sense and antisense strands at 100 μM in water, heating to 95°C and subsequently cooling down to 4°C at a rate of 0.1°C/ min. All DNA was purchased from Life Technologies.

### Equilibrium dissociation constant for homodimerization

Homodimer formation was monitored by following the change in intrinsic tyrosine fluorescence on an SX20 stopped-flow spectrophotometer (Applied Photophysics, Leatherhead, UK). CREB 285–339 (_285_bZIP) in 8 M urea was mixed rapidly in a 1:10 volume ratio with buffer solutions of various (lower) urea concentrations, to achieve protein and urea concentrations outlined in the text. An excitation wavelength of 278 nm was used with a 305 nm cut-off filter, and the temperature was maintained at 25.0°C. At least 50 traces were averaged for a typical measurement.

Kinetic refolding traces were fit using proFit (QuantumSoft, Tomsk, Russia) software to


(1) \begin{eqnarray*}F\left( t \right) = {{F}_0} + {\mathrm{\Delta }}F\left[ P \right]\end{eqnarray*}

where the concentration of monomeric CREB, [P], is given by $[ P ] = \frac{{\frac{{L - 2z}}{4} - ( {\frac{{L + 2z}}{4}} )N{{e}^{ - {{k}_1}zt}}}}{{N{{e}^{ - {{k}_1}zt}} - 1}}$

*L* is the homodimerization equilibrium constant, *k*_1_ is the folding rate constant, $z = \sqrt {\frac{{{{L}^2}}}{4} + 2L{{P}_T}}$, *P_T_* is the total CREB concentration, *N* is $\frac{{\frac{{L - 2z}}{4} + {{{[ P ]}}_0}}}{{\frac{{L + 2z}}{4} + {{{[ P ]}}_0}}}$, and due to the conditions of our refolding experiment ${{P}_0} = {\mathrm{\ }}{{P}_{\mathrm{T}}}$. The equation was derived as shown in the Supplementary Material.

The value of *L* in the absence of urea, and the equilibrium m-value for homodimerization, were extracted as the y-intercept and gradient of a straight line fit of *L* against urea concentration. The value of *k*_1_ in the absence of urea, and the kinetic m-value for folding, were extracted as the *y*-intercept and gradient of a straight line fit of *k*_1_ against urea concentration.

### Circular dichroism

The measurements were carried out for 20 μM protein samples in 10 mM MES pH 6.5, 150 mM NaCl, 10 mM MgCl_2_, 0.05% Tween-20 (biophysical buffer) at 25°C. Spectra were collected using a Jasco J-815 spectropolarimeter equipped with a 1 mm pathlength cuvette and corrected for buffer contribution. Fractional helicities (FH) were calculated using the method described in (56).

### Nuclear magnetic resonance (NMR) spectroscopy and secondary structure propensity (SSP) calculations

NMR experiments were carried out using spectrometers operating at ^1^H frequencies of 600 and 750 MHz. The spectrometers were equipped with Oxford Instruments magnets and Bruker Avance III HD consoles with TCI cryoprobes. Data were acquired using pulse sequences in the TopSpin library from Bruker BioSpin, or using the BEST-TROSY library (57) , with non-uniform sampling for most triple resonance experiments. Data processing was performed using NMRPipe (58), using istHMS for reconstruction of NUS acquired data (59), with visualisation and assignments being performed using CCPN Analysis v2.4.2 (60). Sequential assignments were obtained using 2D and 3D double and triple resonance experiments, including ^1^H-^15^N-HSQC, ^1^H-^15^N-BTROSY, ^1^H-^13^C-HSQC, ^15^N-edited TOCSY-HSQC, ^15^N-edited NOESY-HSQC, HNCO, HN(CA)CO, HNCA, CBCA(CO)NH, HN(CO)CACB, HBHA(CO)NH, HCCH-TOCSY, HCC(CO)NH, and (H)CC(CO)NH. The {^1^H}-^15^N heteronuclear NOE was measured at 25°C in an interleaved experiment recorded with and without ^1^H saturation for 3.5 s at 600 MHz; the {^1^H}-^15^N NOE ratio was calculated as the ratio of the peak intensities in the spectra recorded with and without ^1^H saturation. NMR samples contained 0.3–1 mM ^15^N or ^13^C/^15^N-labelled CREB constructs. The DNA-bound state was examined using a two-fold excess of unlabelled DNA over CREB dimer. Buffer used for NMR was 95% H_2_O/5% D_2_O with 10 mM Tris-d_11_ (Sigma), 150 mM NaCl, 10 mM MgCl_2_, 1 mM NaN_3_ plus protease inhibitors (Pierce™, Thermo Scientific). Spectra were referenced to DSS. Details of assignments for each specific complex are detailed in BMRB depositions 50880, 50872, 50873. Secondary Structure Propensities (SSPs) were calculated using the ^13^Cα and ^13^CO chemical shifts using the SSP program from the Forman-Kay laboratory (61).

### Kinetic measurements of CRE binding and unbinding

Kinetics experiments were carried out in biophysical buffer at 25°C using an SX20 stopped-flow spectrometer (Applied Photophysics). Reaction progress was followed by monitoring the change in fluorescence intensity of the labelled DNA using excitation wavelength of 495 nm and a 515 nm long-pass filter. Association measurements were conducted under the pseudo-first order conditions by rapidly mixing 10 nM DNA with 100–200 nM protein in a 1:1 volume ratio. The averaged traces were fit with a single-exponential decay function to obtain the observed rate constants, and association rate constant (*k*_on_) values were determined from the gradients of linear fits to observed rate constants plotted against protein concentrations. Dissociation measurements were performed by mixing the pre-formed protein-DNA complex (100 and 10 nM, respectively) with a saturating excess of unlabelled competitor CRE DNA (4 μM). For the most severely destabilizing mutations, the concentrations of all reaction components were doubled. Dissociation rate constant (*k*_off_) values were obtained by fitting the averaged traces with a single-exponential decay function. Equilibrium dissociation constants (*K*_D_) for CRE DNA were estimated as the ratios of dissociation to association rates. All data fitting was performed using nonlinear least squares regression analysis implemented in Prism 9 or Prism 10 (GraphPad).

### Φ-value analysis

Φ values were calculated according to (11,12):


(2) \begin{eqnarray*}\Phi = \frac{{\ln \left( {k_{on}^{Ala}/k_{on}^{Gly}} \right)}}{{\ln \left( {K_D^{Gly}/K_D^{Ala}} \right)}}\end{eqnarray*}

where $k_{on}^{Ala}$ and $k_{on}^{Gly}$ are association rate constants for alanine and glycine mutants, respectively, whilst $K_D^{Ala}$ and $K_D^{Gly}$ are equilibrium dissociation constants for alanine and glycine mutants, respectively.

### Analysis of literature Ala-Gly scans

The ratio *k*_on, Gly/Ala_ was calculated as $\frac{{{{k}_{on,\ Gly}}}}{{{{k}_{on,\ Ala}}}}$ for solvent accessible positions in published Φ-value analyses performed with proteins that form helices upon binding. In the case of spectrin *k*_on_ values for mutants were not directly reported and were calculated as *k*_off_/*K*_D_ instead. Errors in the ratio were calculated by propagating the reported errors of the variables (which were themselves based on fitting).

## Results

### Selection of residues for mutation

Previous research, including the published X-ray crystal structure for the CREB-CRE complex, has explored a _285_bZIP peptide corresponding to the fragment of CREB starting with K285 and continuing to K339 (two residues before the natural C-terminus of the protein) (55). Such a fragment contains the whole of the leucine zipper (LZ) motif, as well as the basic region (BR) and is capable of tight binding to the cognate CRE site characterized by low-nM *K*_D_ values. The X-ray structure analysis (PDB ID: 1DH3) confirmed that the most N-terminal residue directly involved in protein-DNA interactions is R286, and that the helical structure of DNA-bound CREB extends over the 285–334 range (46). In performing a Φ-value analysis, it is crucial that the mutations studied do not perturb the binding mechanism (8,62). At the same time, examining as many positions as possible is needed to overcome the uncertainties in interpretation of individual Φ values and substantiate the results. A careful inspection of the X-ray structure of the _285_bZIP-DNA complex revealed that the side chains of the majority of residues that lie within the BR (K285-C310) are involved in the interactions with either DNA or Mg^2+^. The only residues with solvent-exposed side chains are K285, E287, V288, M291, E295, E299, R302 E306 and K309 (Figure 1A, B). K285 was excluded from analysis given its proximity to the N-terminus. The remaining eight residues were subjected to alanine-glycine (Ala-Gly) scanning, a well-established strategy to probe helix formation that minimizes solvation effects of substituting polar amino acids (8,11,12,14–16,20,21,63,64). Each of the solvent-exposed residues was mutated to both Ala and Gly, and the alanine mutant served as a pseudo-wild type to which the corresponding glycine mutant was compared.

### The basic region is disordered but displays limited residual helical propensity

The ensemble of the unbound state in coupled folding and binding processes can contain elements of residual secondary and tertiary structures. The helical predictor AGADIR (65) suggests low levels of helical structure may exist within the DNA binding region of CREB, particularly residues 294–306 (Supplementary Figure S2, black lines). Circular dichroism (CD) measurements (Supplementary Figure S3, black lines) estimate the fractional helicity (FH) of the wild-type _285_bZIP is around 62%, whereas the LZ only constitutes 53% of the residues.

Although CD is very good for observing trends it is notoriously difficult to estimate proportions of different secondary structure accurately. NMR studies can describe residual structure within the unbound state in a more accurate and residue-specific manner. We expressed and purified ^15^N and ^13^C/^15^N labelled versions of _285_bZIP and a related homodimerization-incompetent version with the leucine zipper removed, _285_BR. {^1^H}-^15^N HSQC spectra for the monomeric _285_BR showed narrow dispersion in the ^1^H^N^ dimension typical of a disordered protein whilst that for dimeric _285_bZIP showed more dispersion indicating some folded structure (Supplementary Figure S4). Here we calculate Secondary Structure Propensity (SSP) values for each residue based upon the method developed by the Forman-Kay laboratory (61). Broadly, SSP values of 0 indicate a random coil structure, whilst values of 1 indicate fully helical structure. Monomeric _285_BR appears largely disordered but between 292–300 and 313–315 there are consecutive residues with SSP above 0.2 indicating low levels of helicity within those regions (Figure 1C, bright pink). We further investigated the fast timescale (ps/ns) mobility of the backbone in {^1^H}-^15^N heteronuclear nuclear Overhauser effect (NOE) experiments, and found the peptide to be highly dynamic, particularly towards the termini (Figure 1D, bright pink). The leucine zipper of _285_bZIP appears helical as anticipated, with some helix-fraying towards the C-terminus (Figure 1C, solid black). Interestingly, the SSP within the basic region is also increased considerably over the values found in the monomeric version. Sequence analysis has shown that the spacing between the basic region and leucine zipper is conserved between bZIPs; the region between the final conserved (K/R)(K/R) in the BR to N-terminal leucine of the LZ is 6 amino acids long and has previously been termed a fork or spacer (66,67). SSP in the CREB fork (K305-C310) demonstrates it is neither completely helical nor highly disordered. Instead, it contains significant helical character compared with the N-terminal end of the BR; SSP in the basic region decreases almost monotonically with distance from the leucine-zipper. {^1^H}-^15^N heteronuclear NOE values decrease concomitantly with SSP in the same graded fashion with distance from the zipper indicating increased backbone dynamics – the rigid nature of the zipper itself is demonstrated by values above 0.65 for most of the leucine zipper, with increasing mobility towards the C-terminus where the helicity is lower.

We tested whether the increased helicity in the basic region was due to propagation of helical folding from the LZ, rather than dimerization itself, by inserting four consecutive glycine residues between E306 and Y307 of 285bZIP (_285_bZIP-E306_Y307ins4G). A thermal melt performed by CD demonstrated a cooperative unfolding event at a similar temperature to the wild-type protein (Supplementary Figure S5A) indicating the LZ remains stable in this mutant. Comparative CD spectra demonstrate the predicted significant reduction of helicity expected in the case of propagation, to around 38% (Supplementary Figure S5). This decrease is of a scale consistent with loss of all additional helicity gained by the BR within the bZIP. According to the AGADIR definition of the start of the LZ and the end of the LZ in the X-ray crystal structure 24 out of 60 (40%) of _285_bZIP-E306_Y307ins4G residues would be helical. NMR data are also consistent with the basic region residues being in the same environment as they are in the BR construct (Supplementary Figure S6). As a test of our SSP interpretations we also performed similar experiments for the _285_bZIP incubated with CRE DNA. Upon addition of CRE-DNA the SSP and {^1^H}-^15^N heteronuclear NOE values in the basic region increase still further, reflecting a fully folded and rigid helix which is consistent with the X-ray crystal structure for the CRE-bound complex (Figure 1C and D, dotted black). SSP and NOE results present a very consistent picture for all of the samples, indicating that as helicity is increased fast-timescale backbone dynamics are reduced, and vice versa.

### CREB bZIP forms tight homodimer complexes

**Figure 2.**
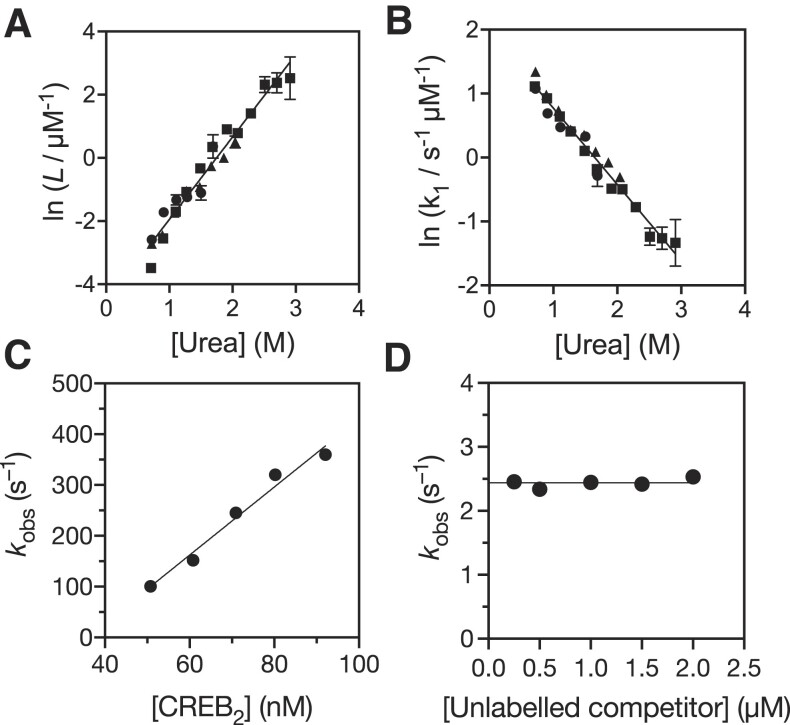
Characterisation of CREB binding interactions. Linear dependence of homodimerization equilibrium constant (**A**) and dimerisation rate constant (**B**) on urea concentration. Extracted values from urea refolding stopped-flow kinetics studies with 2.2 uM (circles) 4.5 uM (triangles) and 9.1 uM (squares) CREB are in good agreement. Straight line fits enable extrapolation to estimate values in the absence of urea. C. Apparent association rate constant for CREB _285_bZIP with 10 nM AlexaFluor®488-CREh depends linearly on CREB _285_bZIP concentration. Concentration is for CREB dimer. Gradient of linear fit (solid line) represents *k*_on_. D. Apparent dissociation rate constant of CREB _285_bZIP from AlexaFluor®488-CREh is independent of concentration of out-competing unlabeled CRE DNA. Average of values represents *k*_off_.

We characterized the homodimerization process using urea refolding kinetic studies to clarify the stoichiometry of CREB in the starting mixtures used in CRE binding studies. We were fortunate to be able to make use of endogenous tyrosine fluorescence as a probe of homodimerisation. CREB was incubated in 8 M urea to favour monomer, and then homodimerisation was promoted by rapid dilution to reduce urea concentrations. Kinetic traces were well fit by an equation (Equation 1) derived for a simple 2-state reversible process $2P \mathbin{{\buildrel\leftharpoonup\over {\smash{\rightharpoondown}}}} {{P}_2}$ (Supplementary Figure S7). In further support of the model the extracted homodimer equilibrium dissociation constant (*L*) and folding rate constant (*k*_1_) both displayed a linear dependence upon final urea concentration that were independent of the CREB concentration (Figure 2A and B). Straight line fits of these data allow estimation of the binding affinity (11 ± 2 nM) and folding rate constant (7.1 ± 0.5 μM^-1^s^-1^) in the absence of urea. The gradients of the lines, or m-values (1.55 ± 0.07 and 0.71 ± 0.03 kcal mol^−1^ M^−1^), reflect changes in the solvent accessible surface area between the monomer and the transition state/dimer (68). The ratio of these two values (0.46) suggests that the transition state is roughly halfway in terms of the folding process.

### Wild-type CREB bZIP associates rapidly with CRE DNA

The binding of _285_bZIP to its cognate site was assessed using a fluorescently-labelled self-annealing oligonucleotide containing a single CRE motif from somatostatin promotor (55,69). Upon rapid mixing of wild-type _285_bZIP with the AlexaFluor®488-CREh DNA by stopped-flow spectroscopy the fluorescence increased. We utilized total CREB concentrations above 100 nM in CREh association studies to probe interactions of dimeric CREB with CRE. Association kinetic traces collected under pseudo-first order conditions fit well to a single exponential decay function (Supplementary Figure S8), with the observed rate constant being linearly dependent on protein concentration (Figure 2C). These observations are consistent with a simple 2-state process for binding of CREB and CREh, without the requirement for dimerization, which is consistent with the majority of CREB being dimeric prior to mixing. The gradient of a straight line fit to the data, (6.7 ± 0.6) × 10^9^ M^–1^ s^–1^, was identified as the fundamental association rate constant (*k*_on_) for dimeric _285_bZIP with its target CREh.

To estimate the dissociation rate constant (*k*_off_) we performed a series of out-competition experiments where pre-formed AlexaFluor®488-CREh·_285_bZIP complexes were rapidly mixed with excess unlabeled CRE DNA. All traces fit well to a single exponential decay function (Supplementary Figure S9), and the observed rate constants were independent of competitor concentration (Figure 2D), which allowed averaging to estimate *k*_off_ as (2.44 ± 0.03) s^–1^. When combined with our *k*_on_ estimate this indicates very tight binding of CREB dimer with CRE (*K*_D_ = (0.36 ± 0.04) nM).

### Mutations in the basic region of _285_bZIP have a marginal impact on residual structure

**Figure 3.**
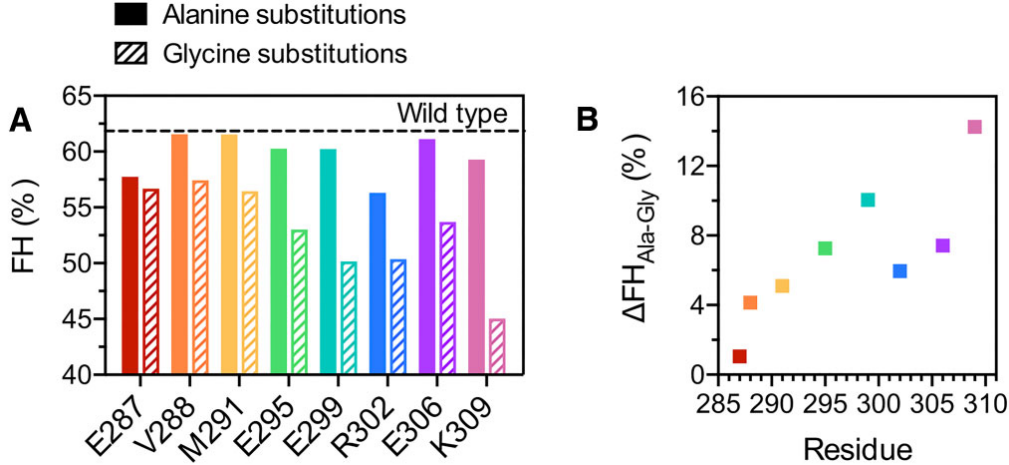
Effects of mutations on residual helicity of CREB _285_bZIP. (**A**) Fractional helicities (FH) of the wild-type CREB _285_bZIP and its mutant versions estimated from the CD response at 222 nm. All alanine mutants are more structured than the corresponding glycine mutants. However, for all the residues except K309, the difference is within 10% of FH. (**B**) The difference in FH between alanine and glycine mutants increases towards the C-terminus of the BR.

All substitutions introduced to the BR of _285_bZIP were predicted to have very marginal effects on helicity by AGADIR (Supplementary Figure S2). However, the sequence-based predictions do not take into account folding within the BR upon dimerization that was observed by NMR. We used CD measurements to estimate the effect of mutation on helicity (Figure 3, Supplementary Figure S3). The FH of the wild-type _285_bZIP is 62%. The alanine substitutions either had no effect or led to a minor decrease in helicity, down to 56% in the case of R302A. Expectedly, all glycine mutants were less structured than the corresponding alanine mutants. The K309G mutant showed a 14% reduction in FH compared to K309A; for the rest of the substitutions the reduction was under 10%. The difference in FH between alanine and glycine mutants exhibited a clear upward trend in the direction of the C-terminus of the BR (Figure 3B).

### Mutation modulates *k*_off_ rather than *k*_on_

**Figure 4.**
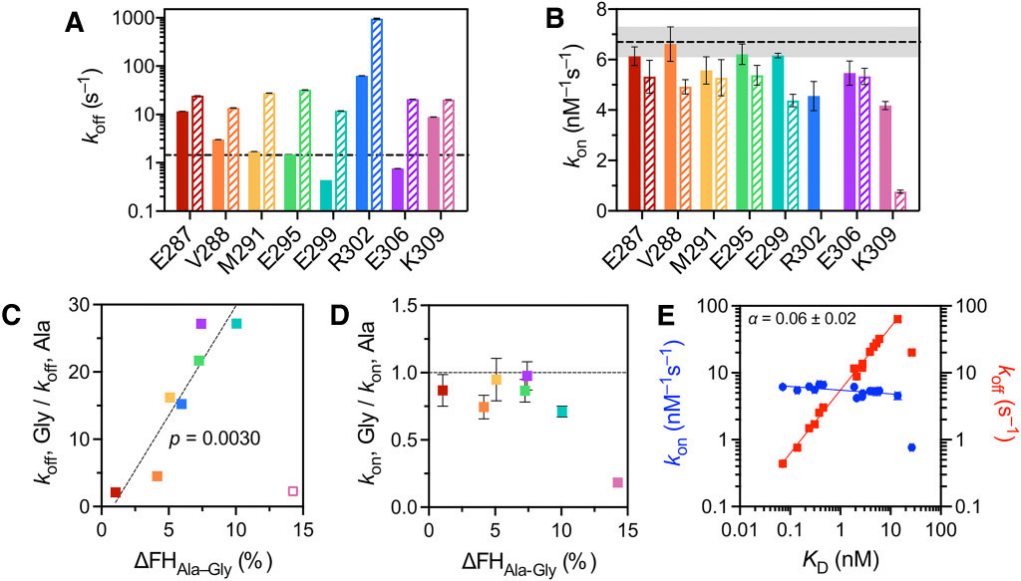
Kinetic effects of mutations in the BR of CREB _285_bZIP. (**A**) Dissociation rate constants (*k*_off_). Alanine mutations are shown as solid bars, glycine mutations are shown as hatched bars, the wild type is shown as a dashed line. (**B**) Association rate constants (*k*_on_) measured in stopped-flow experiments. Designations are identical to (A). The ratios of dissociation rate constants (**C**) and association rate constants (**D**) between each pair of glycine and alanine mutants plotted against the corresponding differences in FH. The decrease in residual structure upon glycine substitutions correlates with a decrease in stability of protein–DNA complex for every mutated residue except K309 (empty square). (**E**) Linear free-energy plot showing *k*_on_ (blue) and *k*_off_ (red) rates against kinetic *K*_D_. Mutant K309G is an outlier.

Dissociation rate constants for the mutant versions of _285_bZIP were estimated as described for _285_bZIP, using a 40-fold excess of unlabelled competitor (Supplementary Figure S10). Most of the alanine substitutions had a considerable effect on dissociation rates (Figure 4A, Supplementary Table S1). Mutations of residues at either ends of the BR resulted in destabilization of the protein–DNA complex as opposed to the stabilizing mutations in the middle of the BR. The only deviation from this pattern was the R302A substitution that demonstrated a remarkable 25-fold increase in *k*_off_ compared to the wild type. All glycine mutants had higher *k*_off_ values than the corresponding alanine mutants (Figure 4A, Supplementary Table S1). The ratio *k*_off, gly_/*k*_off, ala_ correlated well with the difference in FH except for residue K309 (Figure 4C, *P* = 0.003, *n* = 7).

Association kinetics of _285_bZIP mutants were analysed in the same manner as for the wild-type protein (Supplementary Figure S8 and Supplementary Figure S11), although we were unable to directly measure *k*_on_ for R302G due to the significant destabilization caused by the mutation. The association rate constant of _285_bZIP with its target was largely unaffected by alanine mutations (Figure 4B, Supplementary Table S1). The substitutions of R302 and K309 led to a modest decrease in *k*_on_ (ca. 1.5-fold), however, the *k*_on_ value did not increase upon the substitutions of negatively charged residues E287, E295, E299 and E306. The glycine mutants appeared to exhibit either the same or marginally slower association rates than the corresponding alanine mutants, with a prominent exception of K309G that resulted in a 5-fold decrease in *k*_on_ compared to K309A (Figure 4B, Supplementary Table S1). Unlike with *k*_off_ the ratio *k*_on, gly_ /*k*_on, ala_ did not correlate strongly with the difference in FH (Figure 4D).

### The transition state is characterized by low Φ values

**Figure 5.**
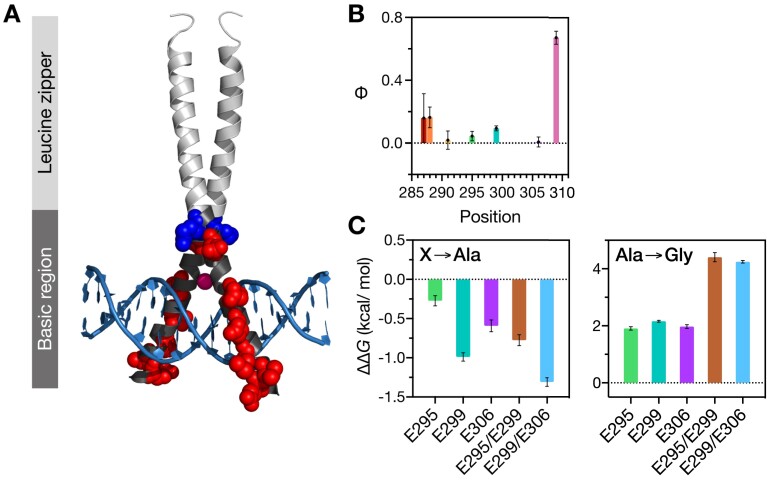
Φ-value analysis of the BR of CREB bZIP. (**A**) Structural positions of residues with the determined Φ values (spheres) in CREB _285_bZIP–DNA complex are coloured red (Φ of 0.0 to 0.2), purple (Φ of 0.2 to 0.6) or blue (Φ of 0.6 to 1.0). The Mg^2+^ ion is shown as magenta sphere. Derived from 1DH3. (**B**) Φ values calculated for alanine-to-glycine mutations in _285_bZIP. The near-zero numbers obtained for nearly all analysed positions give evidence for a disordered transition state. (**C**) The changes in binding free energy (ΔΔ*G*) upon X → Ala and Ala → Gly substitutions. The binding free energies to CRE were calculated from the *k*_off_ and *k*_on_ values. The effects of Ala → Gly substitutions on binding to CRE DNA are additive.

It is informative to consider changes in rate constants in the context of the overall change in stability. Fluorescence-based equilibrium titrations of _285_bZIP with CREh did not yield reliable *K*_D_ values due to the extremely high affinity. We used a kinetic estimate determined from the ratio of *k*_off_ to *k*_on_. As can be seen from a linear free energy relationship (LFER) plot, the effect of mutations on the stability of protein-DNA complex is almost entirely governed by dissociation rate (Figure 4E). The Leffler *α*, calculated as a gradient of log(*k*_on_) versus log(*K*_D_) (11), has a near-zero value of 0.06 ± 0.02, indicating that the transition state far more closely resembles the unbound state than the bound state. The K309G mutant is a clear outlier in the analysis. Structure formation in the transition state was further examined in a more positional manner by calculating Φ values (Equation 2). Seven Φ values were determined in total; each glycine mutant was compared directly with the equivalent alanine mutant to specifically probe helix formation. All the Φ values were low (0.0–0.2), with an exception of K309 which had a value of 0.67 ± 0.04 (Figure 5A, B).

### Double mutations have additive effects on the stability of protein-CRE complex

We selected three residues (E295, E299 and E306) whose Ala-to-Gly substitutions produced the highest changes in binding free energy and analysed the double mutant versions of _285_bZIP with either E295/E299 or E299/E306 being swapped for alanines and glycines. The double glycine mutants appeared to exhibit roughly the same *k*_on_ rates as their double alanine counterparts (Supplementary Figure S12A, B, Supplementary Table S1). However, for both E295G/E299G and E299G/E306G, we observed a more than three orders of magnitude increase in *k*_off_ compared to E295A/E299A and E299A/E306A, respectively (Supplementary Figure S12C, Supplementary Table S1). As a result, the ΔΔ*G* values for double Ala-to-Gly substitutions determined from *K*_D_ comparisons were exceeding 4 kcal/mol, which is approximately the sum of the ΔΔ*G* values determined for the corresponding single mutations (about 2 kcal/mol each) (Figure 5C). Both E295/E299 and E299/E306 yielded near-zero Φ values (0.03 ± 0.04 and −0.022 ± 0.012, respectively).

### Ala-Gly mutation in other helix-forming systems also tends to marginally decrease *k*_on_

**Figure 6.**
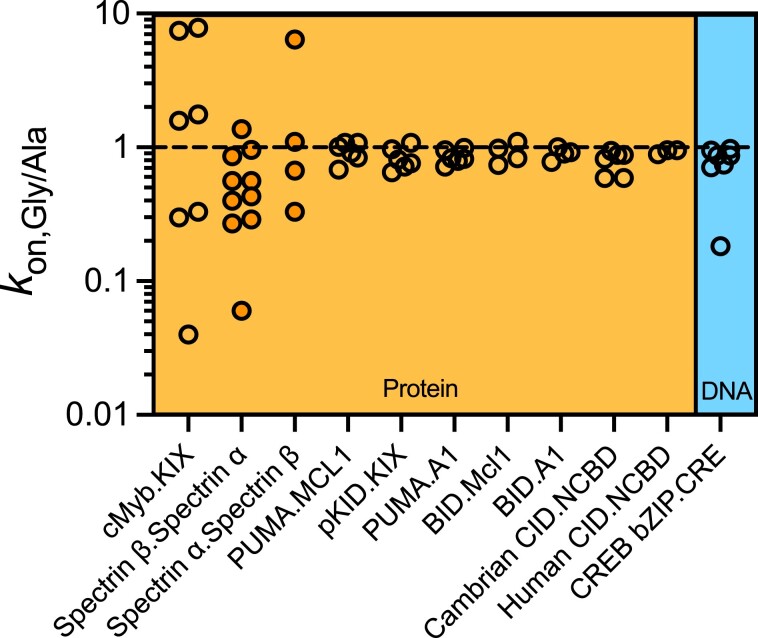
Changes in *k*_on_ resulting from Ala-Gly substitution for disordered proteins that form helices upon binding (9,11–13,20,21). The mutated disordered protein is named first. Transparent circles denote direct estimates of *k*_on_ from pseudo-first order stopped-flow binding studies, whilst orange filled circles denote indirect estimates (calculated using extrapolated *k*_off_ from urea unfolding stopped-flow studies and *K*_D_ from ITC). Nearly all values are below one. Individual values and their errors are displayed in Supplementary Figure S13.

Many proteins that undergo coupled folding and binding form simple helices, including DNA binding domains binding to DNA, and transcriptional activation domains binding to coactivators (1). We found six published kinetic studies where Ala-Gly scanning of disordered proteins that form helices upon binding to proteins had been performed. We calculated *k*_on, Gly/Ala_ ratios for all mutant pairs in these studies (Figure 6). Most mutant pairs from the eight proteins (cMyb (20), α and β spectrin (21), PUMA (9,11), pKID (12), BID (9) and human and cambrian CID (13)) demonstrate similar findings to ours; surface-exposed glycine mutants had consistent or slightly lower *k*_on_ than alanine mutants when binding to partner proteins in 51/56 cases (Figure 6, Supplementary Figure S13). Exceptions were one residue of pKID with a ratio of 1.08 ± 0.06 (12), one residue of spectrin α with a ratio of 6 ± 2 (21), and three residues of cMyb with ratios 7 ± 1, 8 ± 1 and 1.8 ± 0.3 (20). These stated errors are propagated from fitted values rather than replicates and therefore likely underestimate the true error.

## Discussion

### Wild-type CREB kinetic and equilibrium parameters

The bZIP domains are observed to fold upon binding their cognate DNA sequences (2,43,70–72). We have examined the interaction of the isolated bZIP domain of CREB with CRE and find the binding affinity is sub-nanomolar, which is slightly lower than previous estimates for the full-length protein of around 1–2 nM (52,73), but in line with a recent 1 nM upper limit (74). Our results rationalise the observed differences in terms of the experimental approach. The equilibrium dissociation constant for homodimerisation, *L*, is around 10 nM and thus over 10-fold higher than the *K*_D_ for CRE binding. This means that equilibrium approaches to estimate *K*_D_, such as those in the earlier assessments, necessarily provide an *apparent* binding constant that involves a very significant contribution from dimerization. In contrast our kinetic CRE binding experiments are performed at concentrations above *L* and so we probe the interactions of CREB dimer.

The nanomolar value of *L* is also noteworthy because previously reported values differ wildly from tens of μM (75,76) to sub-nanomolar (77). Our value is similar to that found by Bentley et al. for full-length CREB (74) who suggest CREB may be largely dimeric within the cell. For reference cellular CREB concentrations are around 100-fold higher than our reported homodimerisation *K*_D_ (78,79). In determining *L* we revealed that homodimerization was well described as a 2-state process without populated intermediates. The folding rate constant we obtained, of 7 × 10^6^ M^-1^s^-1^, is similar to that reported for GCN4 (17,80), and fairly typical of diffusion-controlled protein-protein interactions for both folded and disordered proteins (81).

The kinetic rate constant for binding to CRE, however, is much higher. The value obtained for *k*_on_, of around 7 × 10^9^ M^-1^s^-1^, is similar to those obtained *in vitro* for other transcription factors with their targets, including GCN4 bZIP (82). Such a high value, around that of the diffusion-limit for uncharged spheres (despite presumed activation energy barriers and geometric constraints), indicates substantial electrostatic rate enhancement. This is presumably a result of complementary charges for the bZIP (+8) and DNA (-30 for AlexaFluor®-488 CREh) providing long-range attractive forces. Apparently consistent with this, removal of a single positive charge by alanine mutation (R302A) does lower *k*_on_. Our observation that removing a single negative charge using the same strategy does not increase *k*_on_ correspondingly implies enhancement is not simply a reflection of the net charges of the molecules however. Mutations of negatively charged residues (E295, E299 and E306) increase the bZIP net charge (from + 8 to + 9 or + 10) but do not increase *k*_on_. All three residues are solvent-facing in the bound state (Figure 1A) and therefore presumably solvent accessible at least some of the time. The choice of a physiologically relevant ionic strength (186 mM) may provide a partial explanation since the Debye length is only around 3 nm in these conditions, however the extremely high *k*_on_ suggests some kind of electrostatic steering is taking place. In contrast the mutation R302A decreases net charge from + 8 to + 7 and does result in a (1.5-fold) decrease in *k*_on_. These observations highlight how rate enhancements do not simply reflect net charges on proteins. An elegant example of this is provided in the KIX system, where *k*_on_ remains enhanced by electrostatics even when the net charge on one partner is reversed (83). One of the most distinctive features of CREB bZIP is a conserved continuous stretch of five positively charged amino acids R301-K305 within the BR (Figure 1A). Analysis of the CRE-bound structure shows that no surface area is buried in the dimer interface for residues 285–303 (Supplementary Figure S14). Although it is not clear exactly how the chains will orient in the absence of CRE, the backbone is more dynamic in this region and helicity will be transient (Figure 1C, D). Taken together it is a fair assumption that at least these residues, which include the first three positively charged residues of the RRKKK motif, are solvent accessible in a sizeable proportion of the unbound ensemble. It is feasible this stretch may play a key role in the initial charge-driven association of protein and DNA via electrostatic steering. Other systems with very significant electrostatic rate enhancements, such as barnase-barstar, also contain highly complementary charged ‘docking’ surfaces as well as high net charges (84).

Residency times for transcription factors on their target promoters must be within useful timescales for successful transcription within eukaryotic cells. Notably the value we obtain for *k*_off_, of around 1 s^-1^, is similar to the 0.42 s^-1^ at which individual fluorescently labelled CREB molecules were observed to leave their long-residency locations in mouse cortical neurons (85). These findings suggest that isolated bZIP domains can encapsulate much of the behaviour of the full-length protein.

### Helical structure is propagated into the basic region in the dimer

NMR analysis showed that higher helical propensities in the C-terminal end of the BR are not present without the folded LZ, and, within the dimer, SSP scores decrease almost linearly with distance from the zipper. Thus, the helical LZ stabilizes helical structure in the neighbouring BR, with the stabilizing effect diminishing with distance i.e. helicity is propagated outwards by dimerization. Single-molecule FRET studies of CREB with fluorescent labels at either end of the LZ (residues 310 and 337) and BR (residues 270 and 310) are consistent with dimerization leading to structural change in the ensemble of the BR (74). Changes to helicity observed by CD for mutant proteins can add further detail. The decrease in FH between each alanine mutant and its corresponding glycine mutant is almost linear with the residue position (Figure 3B) in striking agreement with the spatial pattern of helicity of the apo-state using the more robust NMR method. However, mutation to alanine, a helix favouring residue, causes an appreciable reduction in FH for two residues; E287 and R302. E287 is in a good position to stabilize the helix since its negative charge could offset some of the positive charge towards the N-terminal end of the helix (which would generally interact negatively with the helix dipole). Helix N-capping residues have been demonstrated in GCN4, and suggested within roughly half of bZIP basic regions (86). The crystal structure of the CRE-bound complex shows R302 forming putative salt bridges with E299 and E306 (46), which if present in the unbound state would undoubtedly stabilize helical structure.

Our NMR and Ala-Gly scanning results challenge the concept of a boundary between the BR and LZ in CREB. Historically a fork region between BR and LZ has sometimes been defined in bZIPs (87,88), and helical predictions by AGADIR often show a dip in helicity between the DNA binding region and zipper domain as we observe in the prediction for CREB (Supplementary Figure S2). However, NMR and Ala-Gly scanning have uncovered here an allosteric-like propagation of structure from the adjacent dimerization domain in the dimeric CREB bZIP. This finding implies that the sequence of bZIP ‘forks’ could be functionally very important even when it makes no intermolecular contacts since we reveal that binding affinity depends upon pre-existing helicity propagated through the linker. Fork sequences vary significantly between bZIPs, and whilst predicted size measures for the basic regions of bZIPs are very similar, predicted helicities show much more variation and can also be greatly impacted by exchanging fork sequence (Supplementary Figure S15). However, since these analysis methods exclude the important impact that the LZ can apparently have (Figure 1C, D), residue-specific structural studies would be needed to examine the role of fork sequence identity more robustly.

### The transition state strongly resembles the unbound state

To gain additional insight into the folding and binding mechanism we used alanine and glycine mutants of CREB bZIP in comparative kinetic analyses. Despite binding affinity varying by more than two orders of magnitude between mutants, *k*_on_ values vary very little, with all but one mutant being in the range of 4–7 × 10^9^ M^−1^s^−1^. Instead, there is a very clear and substantive (up to 30-fold) increase in *k*_off_ upon glycine mutation for each Ala-Gly pair (Figure 4A). With the notable exception of K309, there is a good linear correlation between the relative *k*_off_ and change in FH. Thus, stabilizing helical conformations in the BR leads to tighter binding largely through changes in *k*_off_. Indeed, the low Φ values across almost the entire length of the BR (E287–E306) and a near-zero Leffler α imply that the transition state is structurally early and resembles the unbound protein (Figure 4E and Figure 5). The K309G mutant is an outlier in that it does not lie on the straight line revealed on the LFER plot, and the resulting Φ value is considerably higher than the others i.e. this residue is in a largely helical conformation at the transition state. Interestingly our NMR investigation of CREB bZIP indicates that (in the absence of mutation) K309, identified by Uniprot as the final residue of the basic region, is actually largely helical in the unbound state. Given the observed single higher Φ value close to the LZ, and distance-dependent stabilization of BR structure by the helical LZ it is tempting to speculate that folding may initiate from the LZ with further helix formation being stimulated towards the N-terminus in a downhill fashion. The overall level of residual helicity in the BR varies across the bZIP family (43), but this is plausibly a common mechanism for all bZIP dimers. Robustelli *et al.* have previously speculated that GCN4 folding closer to the LZ might stimulate folding further away (89), however this has not been tested.

Φ-value analysis is currently the only experimental method capable of probing the structure of folding transition states, yet it is sometimes criticized because making mutations can make it difficult to adhere to the basic assumptions of the underlying theory. Analysis assumes that mutations introduced to probe the transition state have a negligible effect on the unfolded state (62). To better comply with that requirement, it is ideal to study an IDP with as little residual structure as possible, so that substitutions minimally impact the disordered state of the protein. Our CREB construct does not have high levels of helical structure, but mutations made within the basic region in this study could, and do, alter the unbound (disordered) state somewhat. However multiple lines of evidence point against de-routing effects; changes in protein-DNA complex stability are additive, Φ values remain consistent when calculated in the context of single and double mutants, and the LFER plot is linear (62). Alanine substitutions appeared to produce rather small changes in binding free energy compared to the wild-type _285_bZIP (<1 kcal/mol in magnitude) which also complies with the Φ value requirement for perturbations to be small. Taken together, the _285_bZIP peptide maintains all native protein–DNA interactions while being a convenient target for the Φ-value analysis and therefore represents a good model for studying the mechanism of DNA binding by CREB. Our results are robustly consistent with little to no folding (gain in helical structure) between the unbound and transition state ensembles for folding and binding of CREB with its DNA target CRE.

Most studies for the interaction of the IDPs with protein partners have observed a relatively unstructured transition state. They have proposed either induced fit or a mechanism combining the features of both induced fit and conformational selection (9–13,18–20,90–96). This study explores the mechanism of coupled folding and binding with a DNA partner instead, however our results echo the findings with protein partners in having a very early transition state that strongly resembles the unbound state. Since the transition state is formed after the encounter complex this indicates a heterogenous and dynamic encounter complex as well. This could plausibly facilitate successful and efficient rearrangement from the encounter complex.

### Fishing for fly-casting

One stated advantage to protein disorder is that it may enhance association rate constants over those for folded proteins through a mechanism known as ‘fly-casting’ where encounter rates are accelerated by increased protein capture radii (7). The effect has been implicated in molecular dynamics simulations (97–102), but despite its continued prominence in literature discussion direct experimental evidence for this fly-casting effect is lacking. Enhanced association rates have been observed for mutants of a major histocompatibility complex-like protein, MICA. MICA is mostly folded in its apo-state but has a disordered segment which folds into two helix turns and a loop upon binding its immunoreceptor NKG2D (103). Point mutations aiming to destabilize pre-existing structure of the disordered segment (by weakening stabilizing MICA self-interactions) lead to tighter binding, with 3-fold enhanced *k*_on_ for charge-neutral mutations Q120I and N68W. This could reflect a fly-casting effect, however the mutations are very non-conservative and it may instead reflect destabilization of the unbound state, or a decreased energetic cost for rearrangement from the encounter complex to the transition state. A more targeted experimental investigation is notable in its absence. A capillarity theory for fly-casting revealed that fly-casting is more likely when the binding affinity is high, the barrier small, and the chain can be somewhat rigidly extended towards the target i.e. they suggest a role of residual structure in the unbound state (100). The system investigated here, which meets all these criteria, is therefore an excellent candidate for observing fly-casting experimentally. Indeed the papers outlining the theory highlight protein-DNA binding, contain supplementing simulations of an operator binding to DNA (7,100), and even mention bZIP domains by name (7). Whilst remaining dynamic the BR of CREB has high charge content, and even significant helical structure propagated outwards from the zipper. It has all the hallmarks of an effective fly-casting scenario.

A Φ value like approach to revealing the fly-casting phenomenon (where stability is altered but the binding interface remains unchanged) has been suggested several times (7,100,101). In this work we have done precisely this. Ala-Gly scanning enables us to consider specifically how changes in helicity affect binding kinetics. We have systematically decreased the helical propensity through glycine mutation but we have not observed an increase in *k*_on_. Glycine mutants had smaller or similar *k*_on_ values to their alanine counterparts. In fact the most disordered mutant (K309G) had a significantly lower *k*_on_. Our examination of the results of similar kinetic studies for helix formation upon binding to proteins largely follow a similar pattern, with only five of 56 Ala-Gly pairs having *k*_on, Gly/Ala_ above one. These five values may well reflect errors in *k*_on_. The stated errors are based on propagated fitting errors (rather than replicates), and therefore presumably underestimate the true error. Given the large number of residues examined some outliers are to be expected statistically. Furthermore all but one of these outliers (1.08 ± 0.06 in pKID (12)) are from two datasets that are likely to contain larger errors. In the case of spectrin *k*_on_ were calculated indirectly using *K*_D_ from an individual ITC trace and extrapolated *k*_off_ estimates (21). In the case of cMyb the *k*_off_ values from the linear fits used to extract *k*_on_ were much higher than the robust values obtained from out-competition indicating potential systematic error (20). We note that significantly increasing cMyb residual helicity by another method gave a ratio of 1.1 ± 0.4 upon correction for electrostatics (83). Viewed collectively, mutational studies appear to argue against a general kinetic advantage of protein disorder in target binding.

Since CREB and all of these peptides form helices it is worth reflecting on how the type of structure formed upon folding may impact the effectiveness of fly-casting. Helix formation and especially helix propagation occur within nanoseconds to hundreds of nanoseconds (104,105) (similar or shorter than encounter timeframes (106,107)) maximizing the proportion of successful collisions and therefore *k*_on_ (106). This may be why pre-existing structure does not appear to impact *k*_on_ significantly in these systems. Since an induced fit mechanism is a pre-requisite for fly-casting, and fast folding is expected to assist efficiency of the fly-casting effect (100), helical systems appear to be good fly-casting candidates compared with more complex folds that require contacts between distant residues. However, fly-casting is often described as being due to increased size of disordered proteins compared with folded ones, and these simple helical systems are probably larger when folded. For CREB _285_bZIP the distance between C_α_ for K285 and C310 is 38 Å when fully helical, whereas a 35.5 Å distance is predicted for this sequence by a recent algorithm designed for disordered domains (108). This may explain why no rate-enhancement is observed, but if this is the case then it is worth noting that this will likely not be uncommon for proteins that fold upon binding; many disordered proteins interact with their partners by short linear motifs, of which there are hundreds of classes (109), that fold into extended structures (helices and strands) (110) that will be similarly ‘larger’ than their disordered states. Notably though the kinetic advantage of protein disorder in fly-casting was originally proposed in terms of increased capture radius (7), not increased overall size i.e. radius of hydration. A fully folded protein faces a large entropic barrier because it must approach the binding site within its typical rms displacement (∼ 1 Å) and with suitable orientation that interfacial residues can make contacts. In contrast a disordered protein might ‘start’ binding from any distance within its radius of gyration and dynamically exposes all or most of its residues (7). CD spectra indicate the loss of helical structure from glycine mutation in CREB _285_bZIP is equivalent to up to around 20% of FH in the BR, or roughly half of the pre-existing helicity in that region. Since helicity and dynamics appear to be correlated in this system, we can reasonably infer that dynamics have increased which could increase the capture radius. Capture radii can be accessed in molecular dynamics simulations. The two helix-forming disordered peptide pKID did demonstrate an increased capture radius compared with a ‘pre-structured’ version in molecular dynamics simulations (111). This suggests that capture radius can increase with disorder for helix-forming systems. Yet experimental *k*_on, Gly/Ala_ values for this peptide do not suggest fly-casting (Figure 6, Supplementary Figure S13), and importantly capture rates were also not increased in the simulations (111). The authors pointed out that increased capture radius was more than offset by slower diffusion (111). In reality, the truncated domains used in these studies exist within larger proteins, or even protein complexes, meaning that any diffusional penalty being paid may not be so significant in practice.

It is possible that the suggested technique has not revealed the fly-casting effect because the length of the disordered sequence was not long enough. Within CREB the 6 amino acid fork is immediately next to the consecutive positive charges R301-K305, which should R301-K305 be forming the initial contacts leaves only a short, and fairly structured, segment between. However other parts of the BR could act alone or in concert to initiate the interaction, with subsequent ‘reeling in’ and folding, for example positively charged residues closer to the N-terminus. From this perspective we might consider the entire BR length of 26 amino acids relevant. The other peptides we described have disordered regions of 25 to 55 amino acids, but all form similar amounts of structure (around 5–7 turns of helix in total) (9,11–13,20,21). We found no published suggestions of required lengths for observing fly-casting, but as noted previously the 31 amino acid pKID peptide did display increased capture radius when disordered (111) so this length is presumably sufficient. Finally, our not observing a rate enhancement could be because the changes made in ‘foldedness’ are too small or localized. Unlike the simulations we are not able to make comparisons with a ‘fully folded’ version. Ala-Gly mutations only cause partial disruption of partial helical structure, equivalent of up to around 20% of FH in the BR. The predicted enhanced capture rates from fly-casting are only modest, perhaps 1.4–1.6 fold (7), so the effect size may simply be too small to discern experimentally for fractional changes.

We cannot rule out that the five residues we identified with *k*_on, Gly/Ala_ above one (Supplementary Figure S13) represent particular positions where disorder is advantageous for binding. In molecular dynamics simulations Huang *et al.* did observe higher association rates for more disordered versions of pKID due to a reduced energy barrier to the transition state (111). Recent atomistic MD simulations for another induced fit process also found more disordered encounter complexes were more likely to proceed to bound state (91). In simulations more ordered ligands in encounter complexes have been observed to unfold and refold again during the process of attaining the bound state (91), and there is some limited evidence that disordered proteins may typically have high *k*_on_ even in the absence of electrostatic rate enhancement (112). Nonetheless the five residues appear to be the exception rather than the rule. Any requirement for structure in the transition state, possibly even in induced fit processes, could likewise disadvantage disordered proteins (6). Here Ala-Gly mutations actually lead to statistically significantly decreases in *k*_on_ in five out of seven cases, suggesting that if anything, pre-existing structure accelerates binding rates. The balance between these two requirements will likely depend upon the individual macromolecules, the nature of the binding surface, and the required final topology, and eclipse any kinetic advantage from capture radius i.e. a modest fly-casting effect (7) would be easily overshadowed by energetic terms contained in an exponential.

#### CRE binding within the nucleus

To our knowledge this is the first Φ-value analysis of folding upon binding to DNA, so significantly furthers fundamental mechanistic understanding of protein:DNA interactions, but future binding studies are required to extend understanding into a more biological context. Our study used isolated bZIP domains, which improves the chances of obtaining conclusions general for bZIPs, but neglects some specifics for CREB binding. The rest of the CREB sequence, which contains an acidic activation domain (pKID) and two glutamine-rich domains (28), could modulate DNA binding due to electrostatic and/or steric considerations. A self-interaction between a highly phosphorylated region (the CK cassette) and the bZIP can compete with DNA binding leading to auto-inhibition (113). The nature of the CRE binding transition state, revealed here to strongly resemble the unbound state, may make it robust to such changes, however overall binding constants are likely to be impacted by the surrounding disordered domains. Within the nucleus CREB will also undergo a more complex target binding process. Target search may involve important interactions with non-target DNA including sliding (and potentially intersegmental transfer) which could alter the ‘initial’ CREB structural ensemble that encounters CRE from that of the unbound state. DNA within the eukaryotic nucleus is also found in chromatin and contains modifications such as methylation that can impact binding (28). Our understanding of the influence such factors have on binding mechanisms could be greatly benefited by a bottom-up approach using thoughtfully designed binding and structural studies that consider complications in a systematic fashion.

## Conclusions

Application of Ala-Gly scanning to the folding of CREB upon CRE binding has revealed CREB to have a transition state strongly resembling the structural ensemble of the free protein. The results are consistent with an electrostatically-enhanced binding step followed by fast downhill folding propagating from the already helical leucine zipper. Although we are not able to completely rule out the existence of a fly-casting effect, the suggested experimental approach for testing has yet to reveal evidence for it, questioning its practical relevance. Ultimately what Biology will care about is the rate constant(s), and this is determined by the concentration of the transition state rather than the encounter complex—transition state and electrostatic effects should be expected to dominate over putative capture radius changes in most circumstances.

## Supplementary Material

gkae794_Supplemental_File

## Data Availability

NMR assignments are detailed in BMRB depositions 50880, 50872, 50873.
