# Supplementary material for: The transition state for coupled folding and binding of a disordered DNA binding domain resembles the unbound state
Source: Nucleic Acids Res. 2024 Sep 24;52(19):11822–37. doi: 10.1093/nar/gkae794 (PMC11514473; doi:10.1093/nar/gkae794)
Supplement: gkae794_Supplemental_File [file gkae794_supplemental_file.pdf]

SUPPLEMENTARY INFORMATION for

The transition state for coupled folding and binding of a disordered DNA binding domain resembles the unbound state

Mikhail Kuravsky[1], Conor Kelly[1] Christina Redfield[1] and Sarah L Shammas[1,*]
[1] Department of Biochemistry, University of Oxford, Oxford, OX1 3QU, UK

* To whom correspondence should be addressed.
Tel:+44 1865 613337; Email: sarah.shammas@bioch.ox.ac.uk

SUPPLEMENTARY TABLES

Construct	k_{off} (s^{-1})	k_{on} ($s^{-1}nM^{-1}$)	$K_{D,\,kin}$ (nM)	$\Phi_{Ala\text{-}Gly}$
Wild type $_{285}$bZIP	2.531 ± 0.006	6.7 ± 0.6	0.38 ± 0.03	
$_{285}$bZIP E287A	11.59 ± 0.05	6.1 ± 0.4	1.89 ± 0.11	
$_{285}$bZIP E287G	24.45 ± 0.13	5.3 ± 0.6	4.6 ± 0.6	0.16 ± 0.16
$_{285}$bZIP V288A	3.022 ± 0.009	6.6 ± 0.7	0.46 ± 0.05	
$_{285}$bZIP V288G	13.63 ± 0.08	4.9 ± 0.3	2.77 ± 0.16	0.16 ± 0.07
$_{285}$bZIP M291A	1.708 ± 0.004	5.6 ± 0.5	0.31 ± 0.03	
$_{285}$bZIP M291G	27.7 ± 0.2	5.3 ± 0.7	5.2 ± 0.7	0.02 ± 0.06
$_{285}$bZIP E295A	1.481 ± 0.003	6.2 ± 0.4	0.239 ± 0.016	
$_{285}$bZIP E295G	32.1 ± 0.2	5.4 ± 0.4	6.0 ± 0.4	0.04 ± 0.03
$_{285}$bZIP E299A	0.4379 ± 0.0007	6.16 ± 0.09	0.0711 ± 0.0011	
$_{285}$bZIP E299G	11.90 ± 0.05	4.4 ± 0.2	2.72 ± 0.15	0.09 ± 0.02
$_{285}$bZIP R302A	63.2 ± 0.3	4.6 ± 0.6	13.9 ± 1.8	
$_{285}$bZIP R302G	960 ± 20			n.d.
$_{285}$bZIP E306A	0.7596 ± 0.0012	5.5 ± 0.5	0.139 ± 0.012	
$_{285}$bZIP E306G	20.64 ± 0.13	5.3 ± 0.3	3.9 ± 0.2	0.01 ± 0.03
$_{285}$bZIP K309A	8.85 ± 0.04	4.18 ± 0.16	2.12 ± 0.08	
$_{285}$bZIP K309G	20.18 ± 0.16	0.77 ± 0.06	26 ± 2	0.67 ± 0.04
$_{285}$bZIP E295A/E299A	0.4874 ± 0.0008	4.8 ± 0.4	0.102 ± 0.008	
$_{285}$bZIP E295G/E299G	649 ± 16	3.7 ± 1.0	170 ± 50	0.03 ± 0.04
$_{285}$bZIP E299A/E306A	0.1411 ± 0.0002	3.40 ± 0.07	0.0415 ± 0.0008	
$_{285}$bZIP E299G/E306G	214.1 ± 1.9	4.0 ± 0.3	54 ± 4	-0.02 ± 0.01

Supplementary Table S1. Kinetic and thermodynamic parameters for the DNA binding of all examined $_{285}$bZIP constructs. The errors represent the errors of the fit, or are propagated from errors from fits. n.d.: not determined.

SUPPLEMENTARY FIGURES

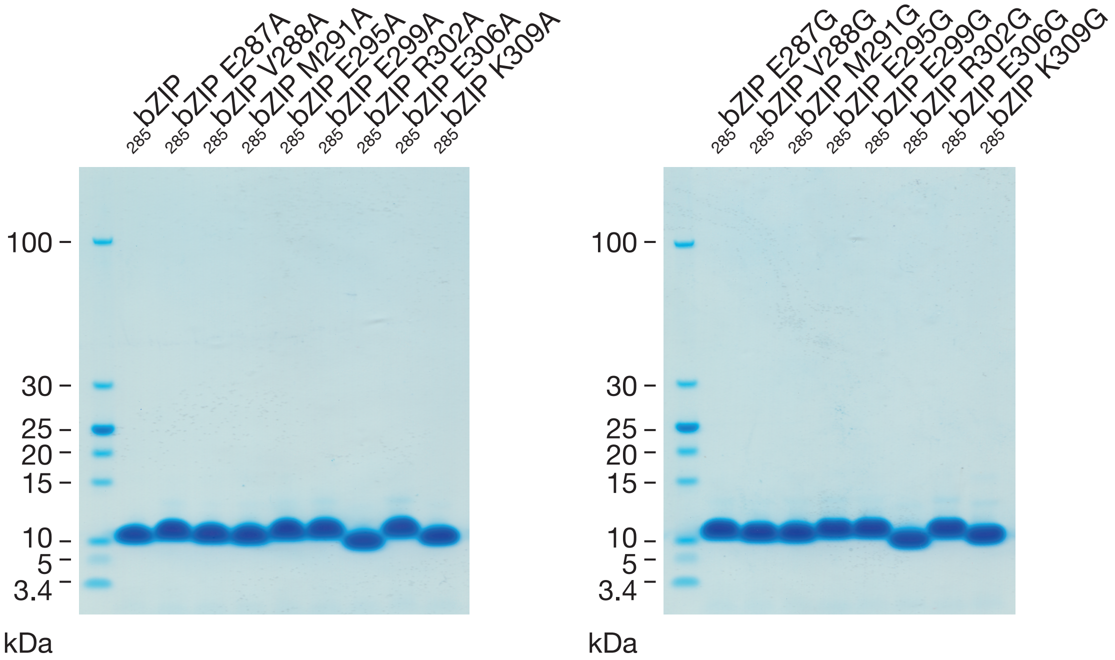

Supplementary Figure 1. SDS-PAGE analysis of CREB $_{285}$bZIP constructs. Molecular weights of the protein standards (first lane) are indicated. Expected molecular weight is 6.7 kDa. Note that disordered proteins typically bind less SDS than globular proteins as a result of their sequence compositions and therefore migrate more slowly in SDS-PAGE analysis*. Precise molecular weight was confirmed by mass spectrometry (ESI-MS). Mutation of positively charged residues R302 and K309 has a minor effect on migration.

* Vladimir N. Uversky and A. Keith Dunker, "Multiparametric Analysis of Intrinsically Disordered Proteins: Looking at Intrinsic Disorder through Compound Eyes", *Analytical Chemistry* **2012** *84* (5), 2096-2104

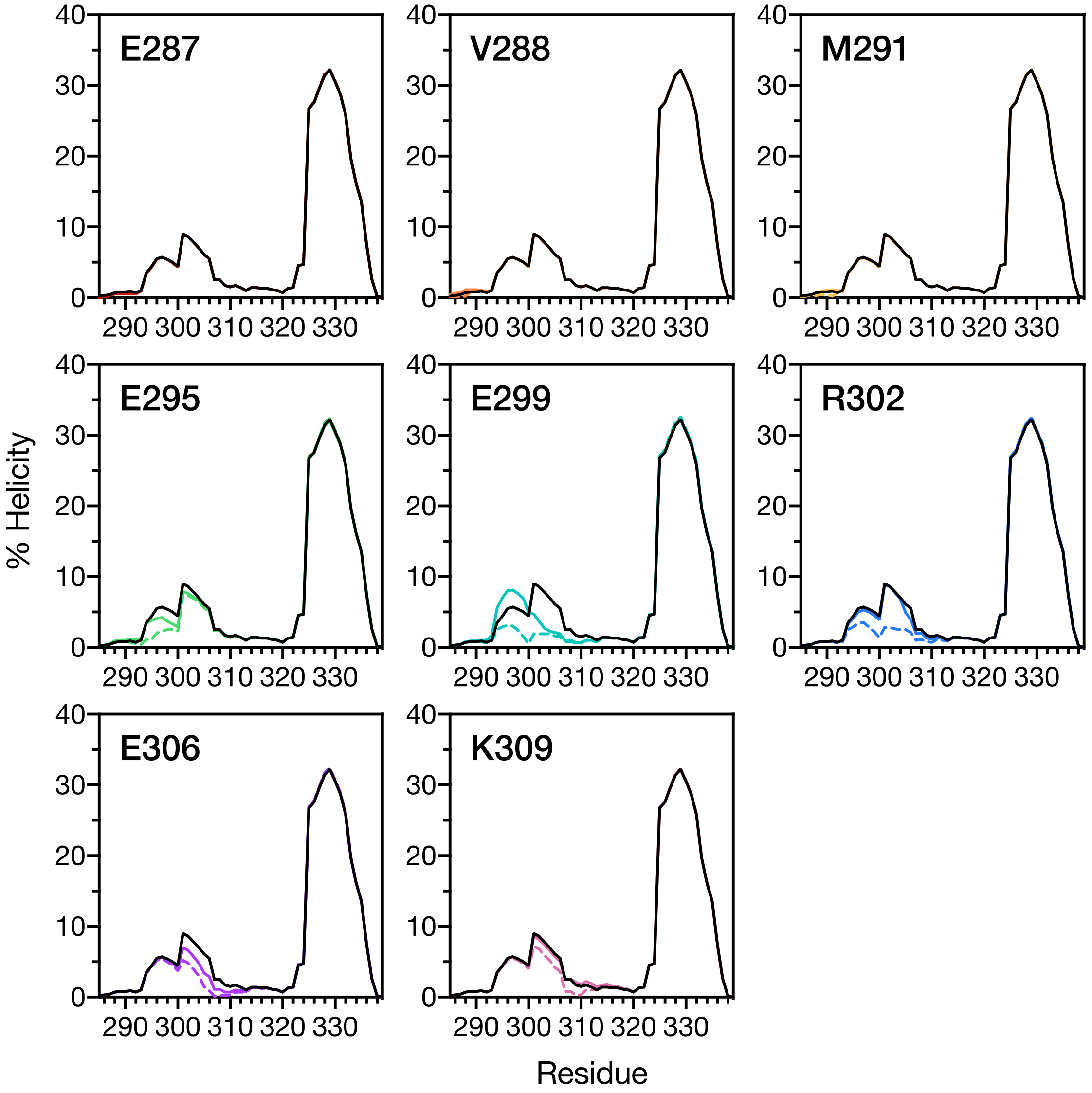

Supplementary Figure 2. AGADIR predictions of helicity based upon amino acid sequence. CREB $_{285}$bZIP sequence predictions are shown as black lines. Predictions for alanine mutants (solid lines) and glycine mutants (dashed lines) are shown in individual panels. Where coloured lines are not visible this is because predictions overlap with CREB $_{285}$bZIP.

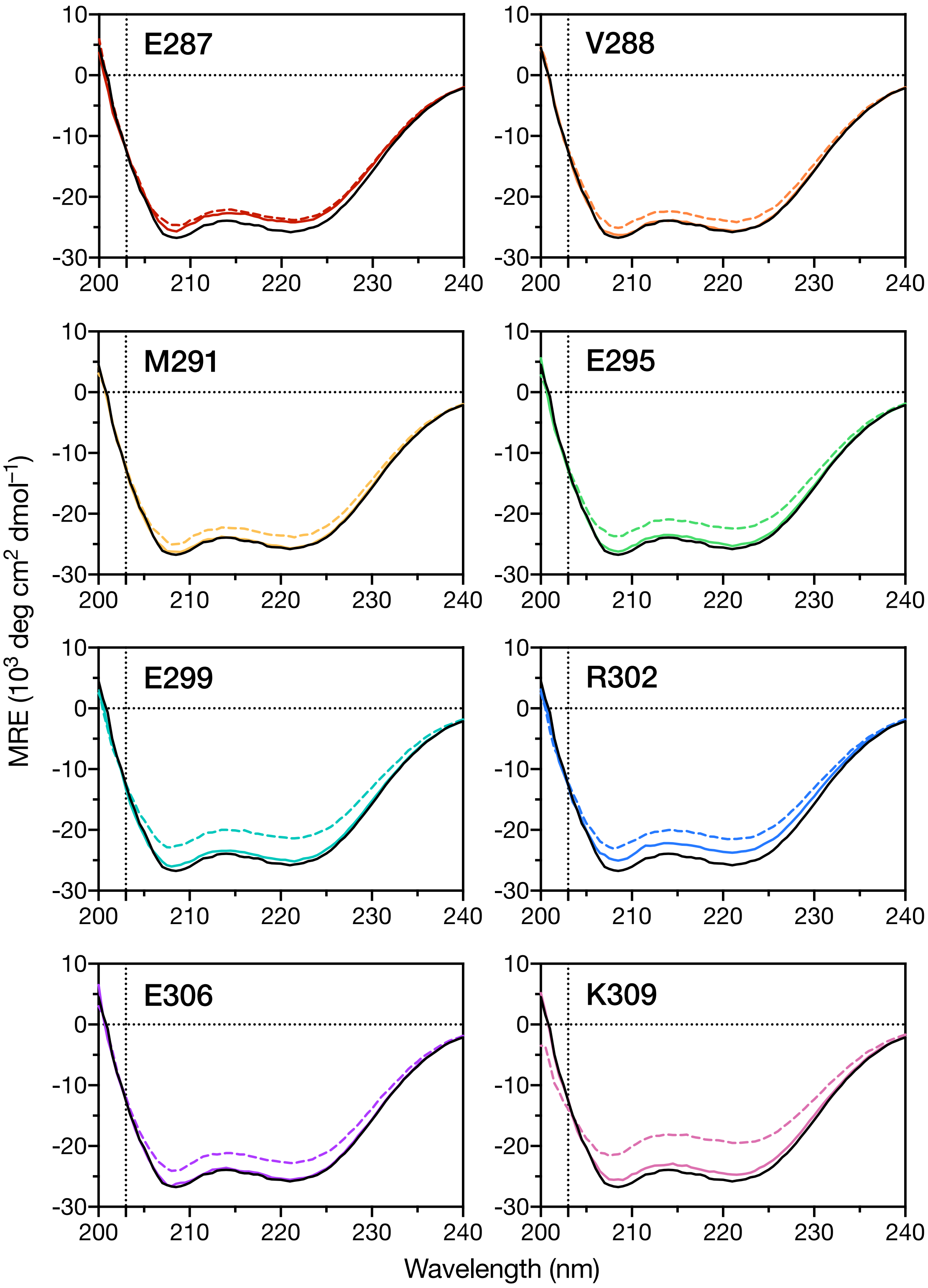

Supplementary Figure 3. Circular dichroism spectra for CREB $_{285}$bZIP and mutants. 20 µM protein samples in 10 mM MES pH 6.5, 150 mM NaCl, 10 mM $MgCl_2$, 0.05% Tween-20 (biophysical buffer) at 25 °C. CREB $_{285}$bZIP is shown as a solid black line, and alanine mutants (coloured solid lines) and glycine mutants (coloured dashed lines) are shown in individual panels. Spectra displayed are an average of three replicates. CD signal at 222 nm can be used as an indicator of helical structure.

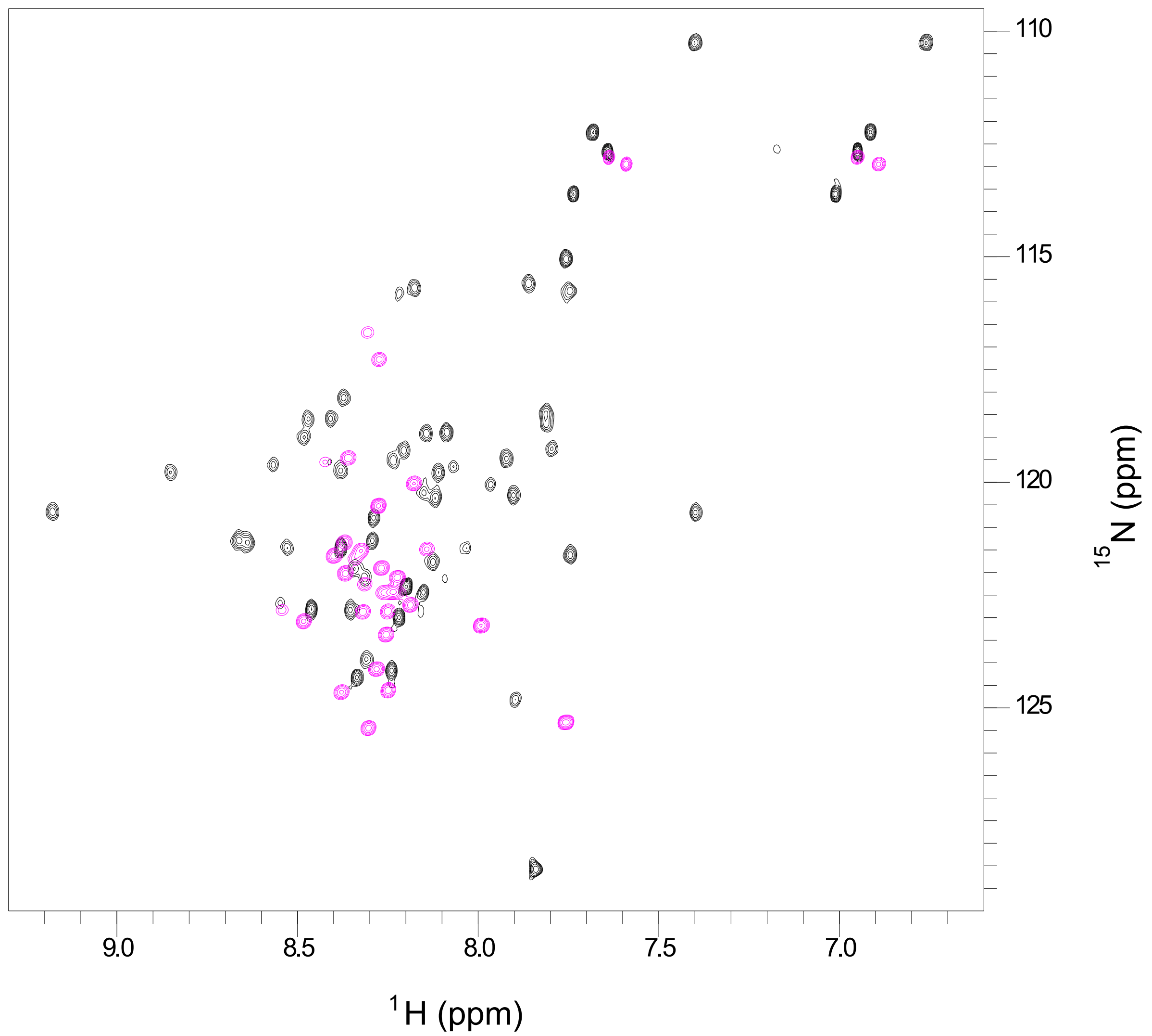

Supplementary Figure 4. Overlay of 600 MHz ^1H-^{15}N HSQC NMR spectra for CREB $_{285}$BR (magenta) and CREB $_{285}$bZIP (black) collected at 25 °C. Samples contained 0.3 -1 mM protein in 95% H_2O/5% D_2O with 10 mM Tris-d_{11} (Sigma), 150 mM NaCl, 10 mM $MgCl_2$, 1 mM NaN_3 plus protease inhibitors (Pierce^{TM}, Thermo Scientific).

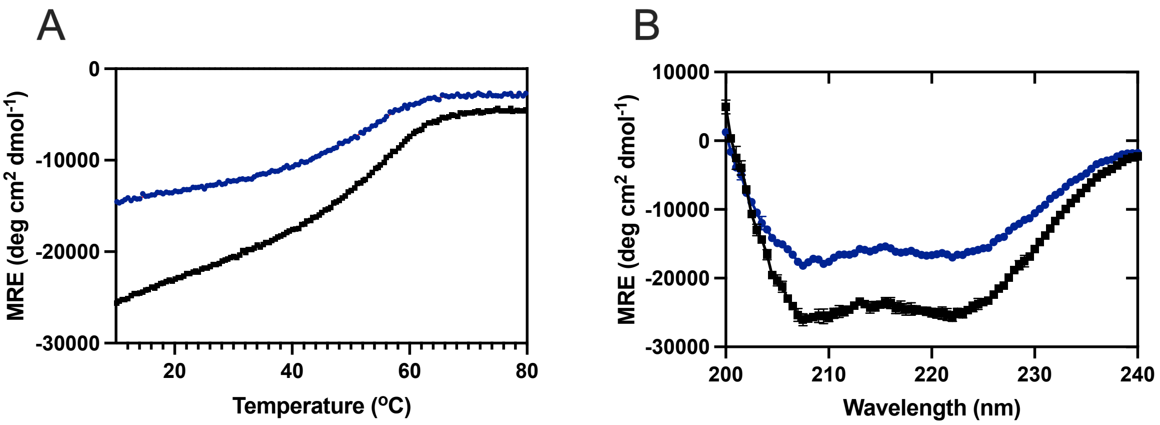

Supplementary Figure 5. A four glycine insert between BR and LZ disrupts helical propagation from the LZ into the BR. A. CD thermal melt demonstrates a cooperative unfolding event for $_{285}$bZIP-E306_Y307ins4G (navy blue) at a similar temperature to $_{285}$bZIP (black). D_{50} are 53°C and 56 °C respectively, consistent with minor destabilisation of the dimer. B. CD spectra demonstrate that $_{285}$bZIP-E306_Y307ins4G (navy blue, 38% helicity) is significantly less helical than $_{285}$bZIP (black, 62% helicity). Data collected in triplicate, error bars represent standard deviation.

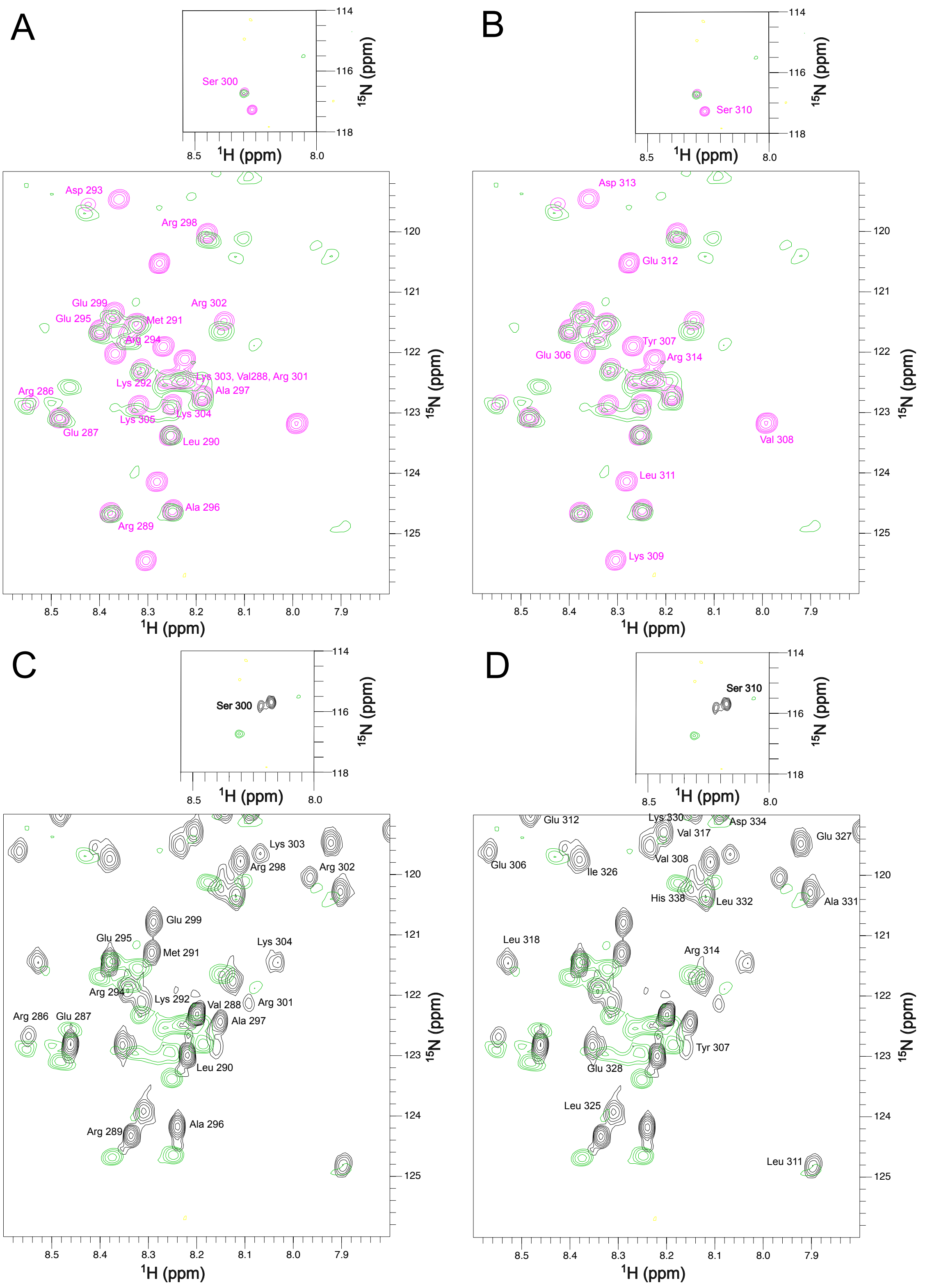

Supplementary Figure 6. A and B. Overlays of 600 MHz ^{1}H-^{15}N HSQC NMR spectra for CREB $_{285}$BR (magenta) and natural abundance HSQC spectrum of CREB $_{285}$bZIP-ins307_GGGG (green). C and D. Overlays of 600 MHz ^{1}H-^{15}N HSQC NMR spectra for CREB $_{285}$bZIP (black) and natural abundance HSQC spectrum of CREB $_{285}$bZIP-ins307_GGGG (green). Peaks assigned for residues 285-305 are shown in panels A and C, and for residues 306-339 in panels B and D. Significant overlay in A, but not in B, indicates similar environments/secondary structure for the basic region residues of the GGGG insertion mutant with the monomeric basic region construct. Samples contained 0.3 -1 mM protein in 95% H_2O/5% D_2O with 10 mM Tris-d_{11} (Sigma), 150 mM NaCl, 10 mM $MgCl_2$, 1 mM NaN_3 plus protease inhibitors (Pierce^{TM}, Thermo Scientific). Data collected at 25 °C.

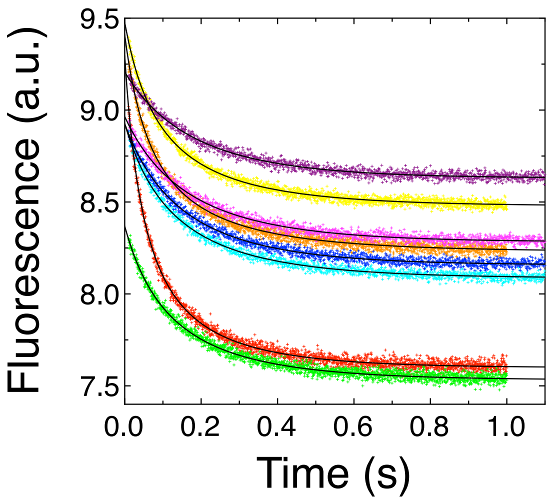

Supplementary Figure 7. Stopped-flow kinetic trace from urea refolding studies. CREB $_{285}$bZIP in 8 M urea was mixed rapidly in a 1:11 ratio to achieve final concentrations of 4.5 μM protein, 0.72 M urea (red), 0.89 M urea (orange), 1.08 M urea (yellow), 1.27 M urea (green), 1.48 M urea (cyan), 1.66 M urea (blue), 1.86 M urea (pink) and 2.04 M urea (purple). Buffers contained 10 mM MES pH 6.5, 150 mM NaCl, 10 mM $MgCl_2$, 0.05% Tween-20 (biophysical buffer) and measurements were performed at 25 °C. Black line is best fit to Equation 1.

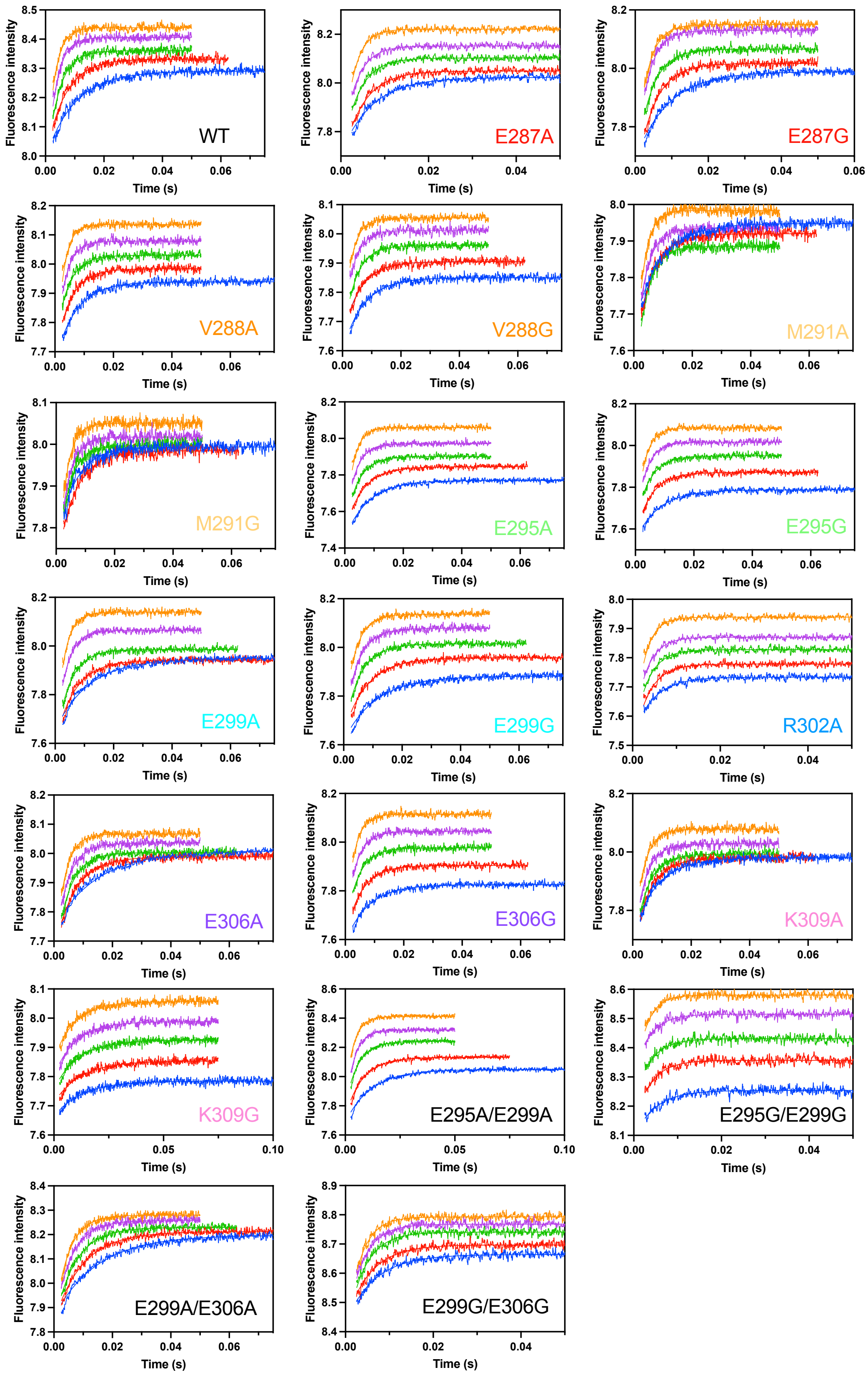

Supplementary Figure 8. Stopped-flow kinetic traces for all CREB $_{285}$bZIP based constructs associating with AlexaFluor®488-labeled CREh. 10 nM AlexaFluor®488-labeled CREh is mixed rapidly with 100 nM (blue), 120 nm (red), 140 nM (green), 160 nM (purple) and 180 nM (orange) CREB $_{285}$bZIP in a 1:1 ratio. Kinetic data are shown as coloured lines, and fitted single exponential decay functions as matching coloured lines. Successive y-axis offsets of 0.05 were applied to fluorescence intensity values to facilitate visualisation. Mutations are stated within the panels. All solutions are in 10 mM MES pH 6.5, 150 mM NaCl, 10 mM $MgCl_2$, 0.05% Tween-20 (biophysical buffer) and measurements were performed at 25 °C.

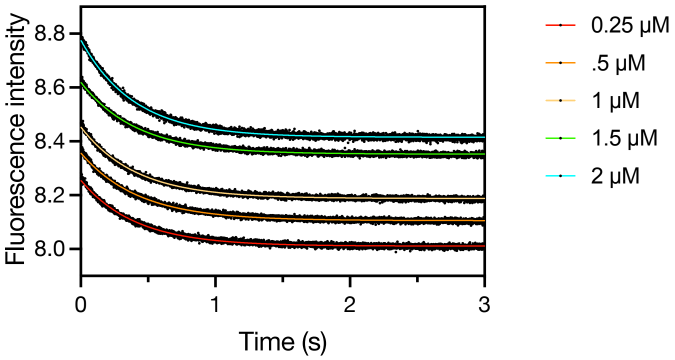

Supplementary Figure 9. Stopped-flow kinetic traces for CREB $_{285}$bZIP dissociation from AlexaFluor®488-labeled CREh. A pre-equilibrated mixture of 10 nM AlexaFluor®488-labeled CREh and 100 nM CREB $_{285}$bZIP was mixed rapidly with 0.25 uM (red), 0.5 uM (orange), 1 uM (yellow), 1.5 uM (green) and 2.0 uM (cyan) unlabelled competitor CRE DNA. Kinetic data are shown as black lines and fitted single exponential decay functions as coloured lines. Successive y-axis offsets of 0.1 were applied to fluorescence intensity values to facilitate visualisation. All solutions are in 10 mM MES pH 6.5, 150 mM NaCl, 10 mM $MgCl_2$, 0.05% Tween-20 (biophysical buffer) and measurements were performed at 25 °C.

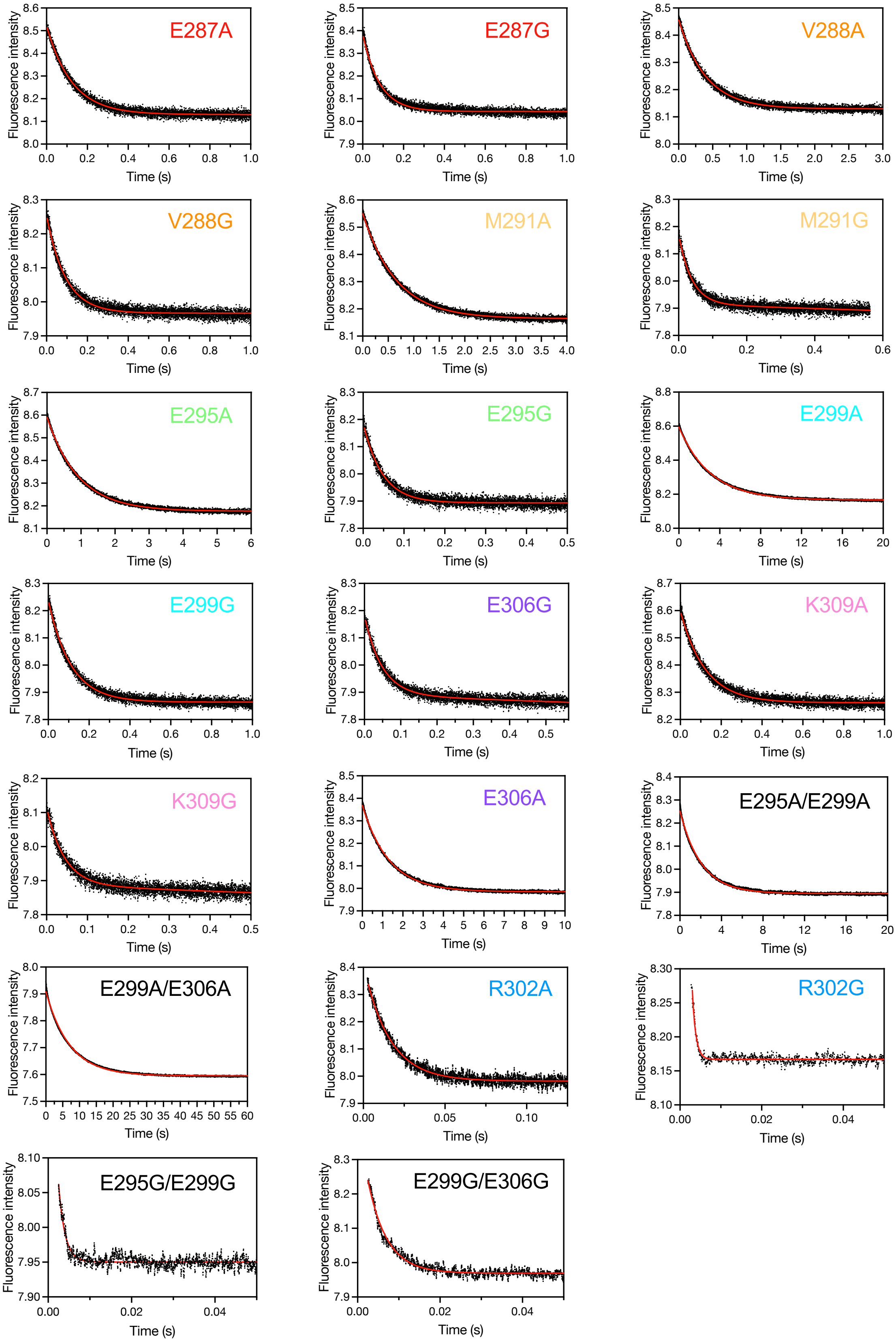

Supplementary Figure 10. Stopped-flow kinetic traces for all CREB $_{285}$bZIP based constructs dissociating from AlexaFluor®488-labeled CREh complexes. Kinetic data are shown as black lines and fitted single exponential decay functions as red lines. A pre-equilibrated mixture of 10 nM AlexaFluor®488-labeled CREh and 100 nM CREB $_{285}$bZIP was mixed rapidly with 4 μM unlabelled competitor CRE DNA in a 1:1 ratio. Mutations are stated within the panels. All solutions are in 10 mM MES pH 6.5, 150 mM NaCl, 10 mM $MgCl_2$, 0.05% Tween-20 (biophysical buffer) and measurements were performed at 25 °C.

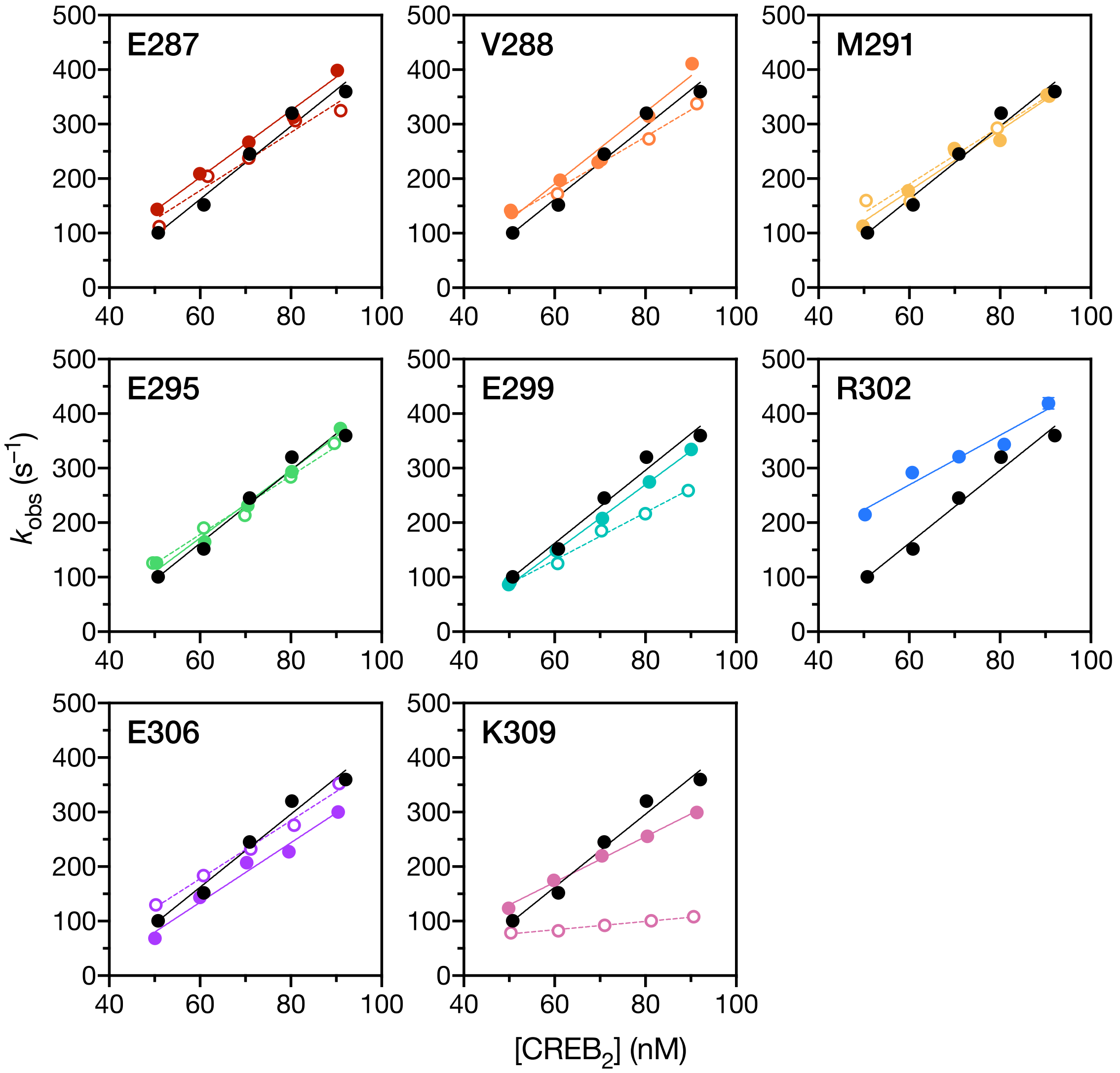

Supplementary Figure 11. Apparent association rate constants with 10 nM AlexaFluor®488-CREh. CREB $_{285}$bZIP is shown as a solid black circles, and alanine mutants (coloured solid circles) and glycine mutants (coloured open circles) are shown in individual panels. Concentrations are for dimeric CREB. Lines are straight line fits, and the gradient represents k_{on}. Kinetic stopped-flow data were collected in 10 mM MES pH 6.5, 150 mM NaCl, 10 mM $MgCl_2$, 0.05% Tween-20 (biophysical buffer) at 25 °C.

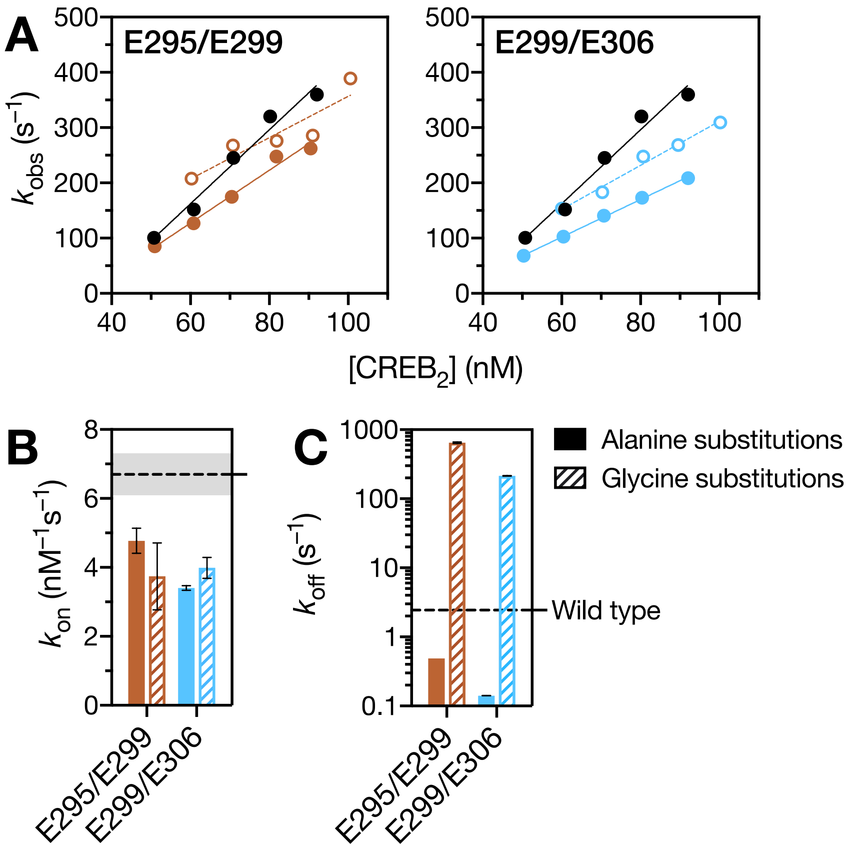

Supplementary Figure 12. Determination of kinetic rate constants for double mutants for double mutant cycle analysis. (A) Apparent association rate constants with 10 nM AlexaFluor®488-CREh for double mutants for residues E295/E299 and E299/E306. CREB $_{285}$bZIP is shown as a solid black circles, and double alanine mutants (coloured solid circles) and double glycine mutants (coloured open circles) for the two position pairs are shown in individual panels. Concentrations are for dimeric CREB. Lines are straight line fits and extracted rate constants (gradients) are shown in (B). (C) Dissociation rate constants for double mutants. Wild-type value is indicated by dotted line. Kinetic stopped-flow data were collected in 10 mM MES pH 6.5, 150 mM NaCl, 10 mM $MgCl_2$, 0.05% Tween-20 (biophysical buffer) at 25 °C.

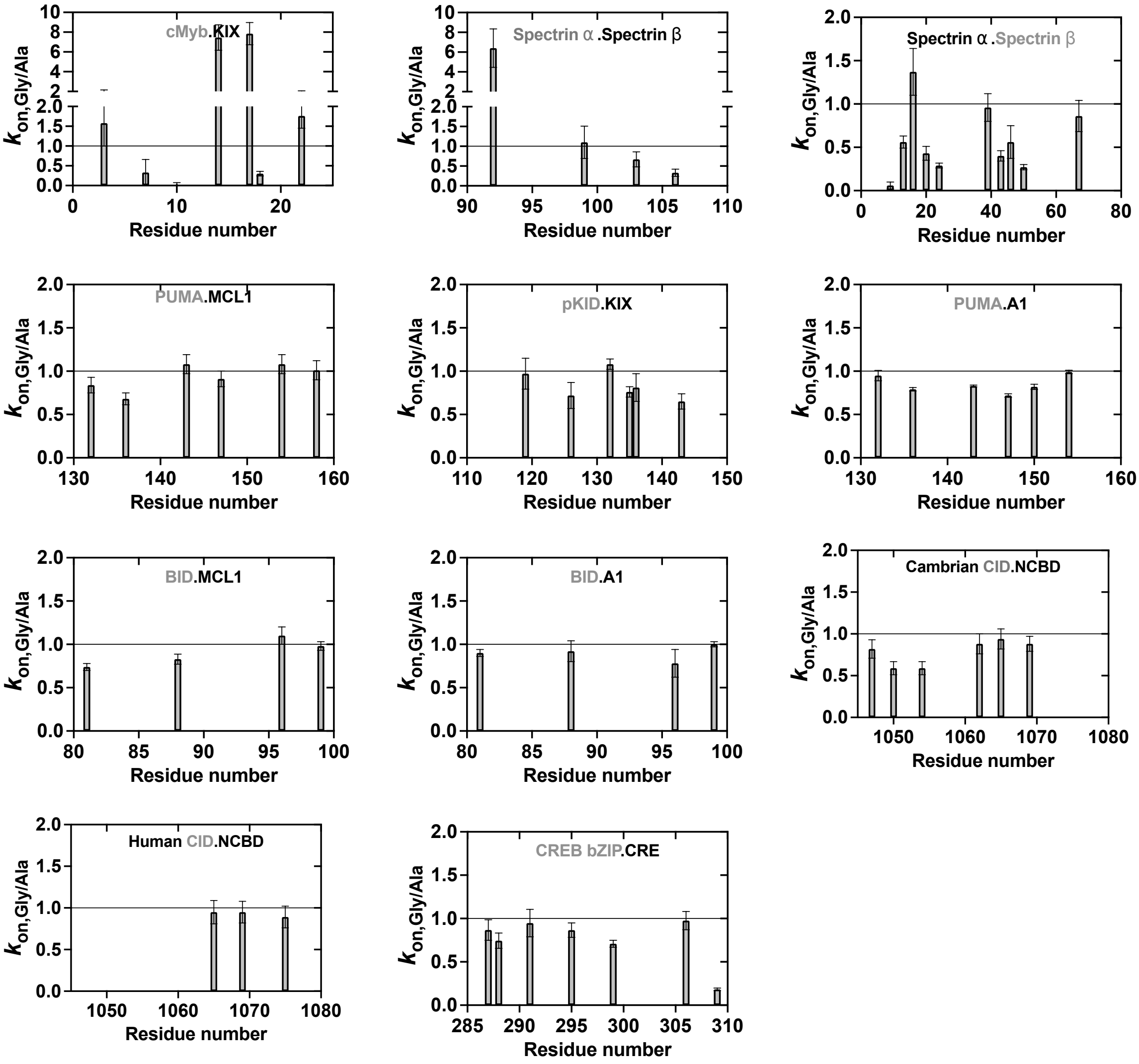

Supplementary Figure 13. Analysis of existing kinetic Ala-Gly scans for proteins that form helices upon binding to their partners. Residue specific values of $k_{on,Gly/Ala}$ are shown for each protein, including the standard error on the mean (calculated by propagation of fitting errors). The name of the mutated disordered protein is shown in grey in the panel title. k_{on} values were identified as the gradient of straight line fits to pseudo-first order association kinetic data, with the exception of spectrin. For spectrin k_{on} were determined indirectly as $k_{off}K_a$; k_{off} were extrapolated to 0M urea from urea unfolding kinetics and K_a were determined from a single ITC trace. References for each study are found in the main text.

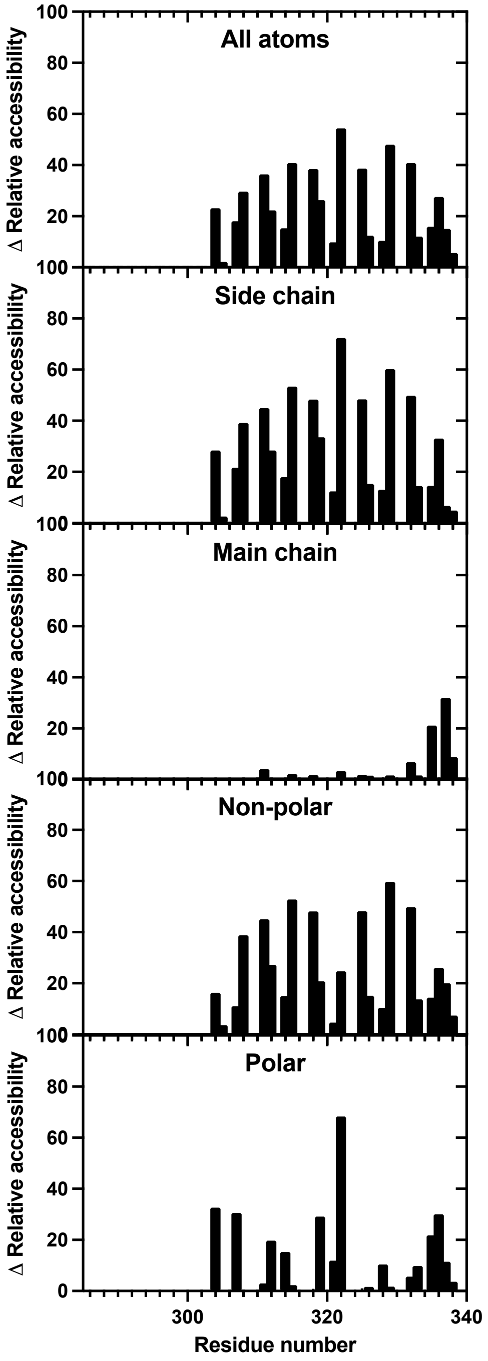

Supplementary Figure 14. Change in solvent accessibility for each residue of CREB $_{285}$bZIP in the CREB $_{285}$bZIP.CRE complex (PDB: 1DH3) due to homodimer formation (relative to total possible for amino acid type). The difference in SASA for each residue was calculated as the difference between the SASA when both CREB chains were present, and the average SASA for a single CREB chain. Calculations were performed in naccess (http://www.bioinf.manchester.ac.uk/naccess/).

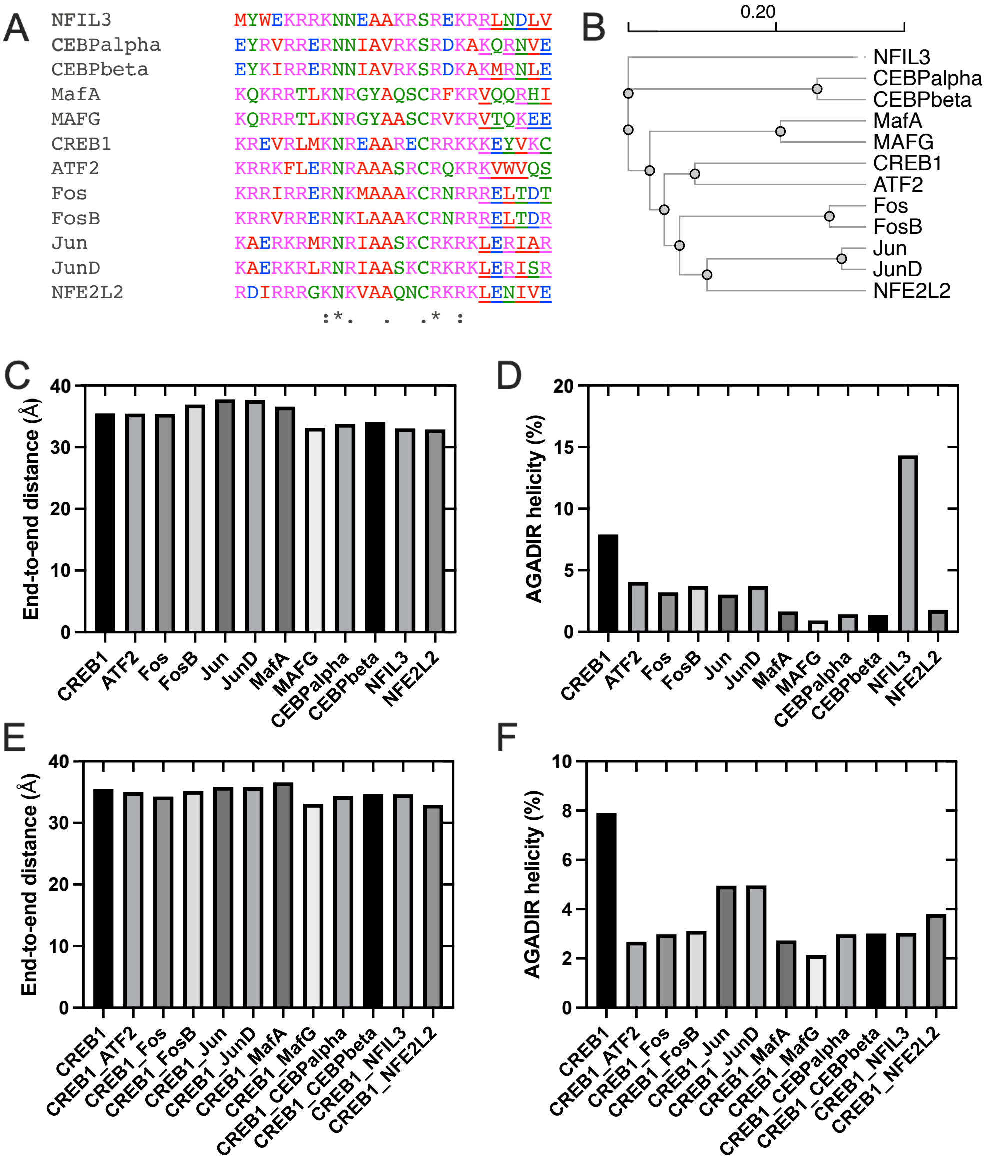

Supplementary Figure 15. A. Basic regions of all human bZIPs with published structures aligned together. Fork sequences are underlined. B. Guide tree from Clustal Omega (https://www.ebi.ac.uk/jdispatcher/msa/clustalo) for the cropped protein sequences aligned in A is included as a rough indicator of sequence similarity of examined basic regions. It is not intended as an evolutionary tree. Predicted end-to-end distances (C) and helicities (D) for the sequences in A. The natural CREB fork sequence within the CREB sequence was replaced with fork sequences from each of the other proteins, resulting in predicted end-to-end distances (E) and helicities (F). Whilst end-to-end distances vary little between constructs, the helicity varies more significantly. Predictions were made with Albatross (https://github.com/holehouse-lab/ALBATROSS-colab) and AGADIR (http://agadir.crg.es/).

SUPPLEMENTARY METHODS

Derivation of equation for fitting homodimer refolding studies

Let P denote the protein monomer and P_2 – the protein dimer. The simplest chemical equation modelling their equilibrium is

$$2P \leftrightharpoons P_2. \qquad \text{(Eq. S1)}$$

Let $[P]$ and $[P_2]$ denote the concentrations of the respective species, and P_T denote the total concentrations of protein. Then mass balance gives

$$P_T = [P] + 2[P_2]. \qquad \text{(Eq. S2)}$$

Let L denote the equilibrium dissociation constant. When the system reaches equilibrium,

$$L = \frac{[P]^2}{[P_2]}. \qquad \text{(Eq. S3)}$$

Let k_1 and k_2 denote the association and dissociation rate constants, respectively, for homodimerization reaction Eq. S1. The rate of change in monomer concentration can be expressed as:

$$\frac{d[P]}{dt} = -k_1[P]^2 + 2k_2[P_2]. \qquad \text{(Eq. S4)}$$

At equilibrium $\frac{d[P]}{dt} = 0$, combining this with Eq. S3. shows the equilibrium constant L is

$$L = 2\frac{k_2}{k_1} \qquad \text{(Eq. S5)}$$

and requiring mass balance (Eq. S2) leads to:

$$-k_1 dt = \frac{d[P]}{[P]^2 + \frac{L}{2}[P] - \frac{L}{2}P_T}. \qquad \text{(Eq. S6)}$$

Finding the roots of the quadratic expression in the denominator allows rewriting as:

$$-k_1 dt = \frac{d[P]}{\left([P] + \frac{L+2z}{4}\right)\left([P] + \frac{L-2z}{4}\right)}, \quad \text{where} \quad z = \sqrt{\frac{L^2}{4} + 2LP_T} \qquad \text{(Eq. S7).}$$

Eq. S7. may then be integrated to obtain an expression for the concentration of monomeric protein with time:

$$[P] = \frac{\frac{L-2z}{4} - \left(\frac{L+2z}{4}\right)Ne^{-k_1 zt}}{Ne^{-k_1 zt} - 1}, \qquad \text{(Eq. S8)}$$

where N is a constant of integration that may be written in terms of the initial monomer concentration $[P]_0$ by evaluation of Eq. S8 at time zero:

$$N = \frac{\frac{L-2z}{4} + [P]_0}{\frac{L+2z}{4} + [P]_0}. \qquad \text{(Eq. S8a)}$$
